# Distinct roles for type I and type III interferons in virulent human metapneumovirus pathogenesis

**DOI:** 10.1371/journal.ppat.1011840

**Published:** 2024-02-05

**Authors:** Yu Zhang, Jiuyang Xu, Margot Miranda-Katz, Jorna Sojati, Sharon J. Tollefson, Michelle L. Manni, John F. Alcorn, Saumendra N. Sarkar, John V. Williams

**Affiliations:** 1 Department of Pediatrics, University of Pittsburgh School of Medicine, Pittsburgh, Pennsylvania, United States of America; 2 Tsinghua University School of Medicine, Beijing, China; 3 Department of Pediatrics, Vanderbilt University Medical Center, Nashville, Tennessee, United States of America; 4 Department of Microbiology & Molecular Genetics, University of Pittsburgh School of Medicine, Pennsylvania, United States of America; 5 Institute for Infection, Inflammation, and Immunity in Children, University of Pittsburgh, Pennsylvania, United States of America; Icahn School of Medicine at Mount Sinai, UNITED STATES

## Abstract

Human metapneumovirus (HMPV) is an important cause of acute lower respiratory infection in children and adults worldwide. There are four genetic subgroups of HMPV and both neutralizing antibodies and T cells contribute to protection. However, little is known about mechanisms of pathogenesis and most published work is based on a few extensively passaged, laboratory-adapted strains of HMPV. In this study, we isolated and characterized a panel of low passage HMPV clinical isolates representing all four genetic subgroups. The clinical isolates exhibited lower levels of *in vitro* replication compared to a lab-adapted strain. We compared disease phenotypes using a well-established mouse model. Several virulent isolates caused severe weight loss, lung pathology, airway dysfunction, and fatal disease in mice, which was confirmed in three inbred mouse strains. Disease severity did not correlate with lung viral titer, as virulent strains exhibited restricted replication in the lower airway. Virulent HMPV isolates were associated with markedly increased proinflammatory cytokine production and neutrophil influx; however, depletion of neutrophils or genetic ablation of inflammasome components did not reverse disease. Virulent clinical isolates induced markedly increased type I and type III interferon (IFN) secretion *in vitro and in vivo*. STAT1/2-deficient mice lacking both type I and type III IFN signaling showed reduced disease severity and increased lung viral replication. Inhibition of type I IFN signaling using a blocking antibody or genetic ablation of the type I IFN receptor reduced pathology with minimal effect on viral replication. Conversely, blockade of type III IFN signaling with a neutralizing antibody or genetic ablation of the IFN-lambda receptor had no effect on pathogenesis but restored viral replication. Collectively, these results demonstrate distinct roles for type I and type III IFN in HMPV pathogenesis and immunity.

## Author summary

Human metapneumovirus (HMPV) is a leading cause of acute lower respiratory infection in children and adults worldwide. However, little is known about mechanisms of pathogenesis and most published studies use a few laboratory-adapted strains of HMPV. In this study, we isolated and characterized disease phenotypes of a panel of HMPV isolates. We identified several virulent isolates capable of causing severe and fatal disease in mice. Virulent HMPV isolates were associated with markedly increased proinflammatory cytokine production as well as increased type I and type III interferon (IFN) secretion *in vitro* and *in vivo*. Inhibition of type I IFN signaling reduced pathology with minimal effect on viral replication. Conversely, blockade or genetic ablation of type III IFN signaling had no effect on pathogenesis but restored viral replication. Collectively, these results demonstrate distinct roles for type I and type III IFN in HMPV defense and disease.

## Introduction

Human metapneumovirus (HMPV) is a pneumovirus discovered in 2001[1] that is a leading cause of acute lower respiratory infection in children and adults worldwide, often second only to respiratory syncytial virus (RSV)[2–8]. Epidemiologic host factors associated with disease severity include premature birth, asthma or chronic obstructive pulmonary disease, immunocompromise, and older age [5,7–14]. HMPV is a serious pathogen among immunocompromised adults and children with AIDS, cancer, hematopoietic stem cell transplant, or solid organ transplant [15–18]. However, little is known about mechanisms of HMPV pathogenesis. Most published studies use a few laboratory-adapted strains of HMPV, which may not be as representative of human disease in models such as mice, hamsters, cotton rats, and non-human primates [19–24]. The most common mouse strains used are BALB/c and C57BL/6 (B6); DBA/2 exhibits high viral replication, but B6 offers substantially more genetically altered strains [20,22,23,25–27].

Recent studies of the related respiratory syncytial virus (RSV) showed that RSV clinical isolates induce higher mucin production and more severe respiratory disease in mice, and this virulence mapped to the fusion (F) protein [28–30]. Another study found that virulent RSV clinical isolates were correlated with a distinct pattern of innate immune activation [31]. HMPV, like other viruses, is capable of inhibiting type I interferon (IFN) signaling, though the mechanisms are not fully defined, and multiple viral proteins have been implicated including glycoprotein (G), small hydrophobic (SH), and the M2-2 protein [32–37]. Nonetheless, type I IFN contributes to disease pathogenesis [38,39]. One prior study reported that HMPV induced type III IFN and that treatment of mice with exogenous type III IFN reduced viral titer [40]. However, the roles of type I and III IFN during HMPV infection have not been fully elucidated.

In this study, we isolated and characterized disease phenotypes for a panel of HMPV isolates covering all four genetic subgroups. We identified several virulent isolates capable of causing severe and fatal disease in mice. Virulent HMPV isolates were associated with markedly increased proinflammatory cytokine production as well as increased type I and type III IFN (IFN) secretion *in vitro* and *in vivo*. Inhibition of type I IFN signaling reduced pathology with minimal effect on viral replication. Conversely, blockade or genetic ablation of type III IFN signaling had no effect on pathogenesis but partially restored viral replication. Collectively, these results demonstrate distinct roles for type I and type III IFN in HMPV pathogenesis and immunity.

## Results

### Isolation and identification of HMPV clinical isolates

A panel of nine HMPV clinical strains were isolated between 1985–2004 from patients with acute respiratory illness in Nashville, TN, or Cincinnati, OH (**Table 1**). The clinical isolates were grown in LLC-MK2 cells, maintained below 10 passages, and sucrose purified prior to full genome deep sequencing. There were no sequences from other pathogens in the isolates, and individual testing by real-time RT-PCR was negative for adenovirus; influenza A, B, and C; coronaviruses 229E and OC43; parainfluenzaviruses 1–4; RSV; rhinoviruses; and enterovirus D-68 and all were negative [41–43]. We performed clustering analysis on the clinical isolates based on their full-length N, F, and G gene sequences, together with the published sequences of reference strains NL/1/00, NL/1/99, and CAN/97-83. The phylogenic trees generated by N, F, and G genes were similar (**S1A–S1C Fig**). The genomic sequences of TN/94-49 and TN/94-66 were nearly identical, as were 1501 and 1509; these may represent the same strains infecting multiple patients in the same season. We calculated the nucleotide and amino acid identities of the nine viral proteins. Similar to previous observations for other HMPV sequences [44–47], the N, P, M, F, M2-1, M2-2 and L genes were highly conserved at both nucleotide and amino acid level (**S1 Table**). The SH and G genes were less conserved with amino acid identity even lower than nucleotide identity, suggesting low selection pressure on these regions.

**Table 1:** Strain information of HMPV Clinical Isolates.

Strain	Subgroup	Year of Isolation	Site	GenBank^a^
TN85-416 (42)	A1	1985	Nashville, TN, USA	PP086008
TN94-344 (1501)	A1	1994	Nashville, TN, USA	PP086535
TN94-711 (1509)	A1	1994	Nashville, TN, USA	PP086007
TN/94-49	A2	1994	Nashville, TN, USA	JN184400
TN/94-66	A2	1994	Nashville, TN, USA	PP086006
C1-334	B1	2004	Cincinnati, OH, USA	KC562242
C2-202	B1	2004	Cincinnati, OH, USA	KC562235
TN/91-320	B2	1991	Nashville, TN, USA	KC403972
TN/96-35	B2	1996	Nashville, TN, USA	JF929882

^a^All sequences have now been submitted to GenBank and the numbers are listed in the table. See also S1 Table and S1 Fig for more information on HMPV clinical isolates.

We tested *in vitro* growth kinetics in LLC-MK2 cells of 4 strains, each representing a unique genetic subgroup. All four isolates exhibited log growth at 1–3 days post inoculation and virus titer decreased in supernatant after 7 days (**S1D Fig**). However, the four isolates reached different peak titers; the laboratory strain TN/94-49 replicated to ~10^7^ PFU/ml, but clinical isolates C2-202, TN/91-320, and 1501 only reached 10^5^−10^6^ PFU/ml. The clinical isolates also exhibited cytopathic effect (CPE) at various degrees. HMPV TN/94-49 cause widespread syncytium formation and cell detachment at 5 days post infection (**S1E Fig**). The monolayers of 1501, C2-202, and TN/91-320 infected cells remained intact with minimal syncytia. As previously shown, these isolates had different abilities to form plaques under agarose overlay in LLC-MK2 cells [48].

### HMPV clinical isolates cause differential weight loss and disease in mice

**Fig 1:**
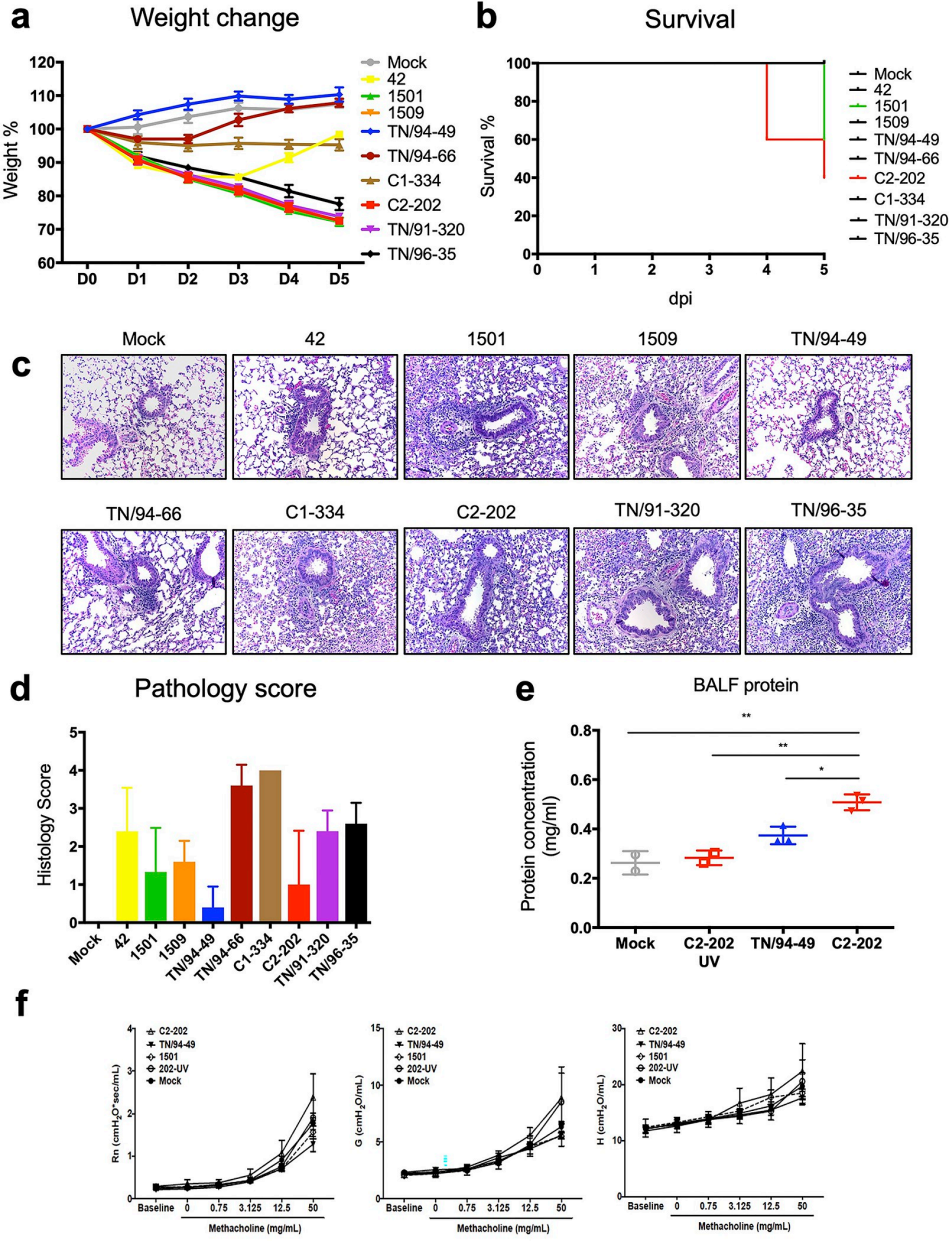
HMPV clinical isolates cause variable disease in mice. (a, b) Seven-week old female B6 mice were infected with 1.0 x 10^6^ PFU HMPV clinical isolates or LLC-MK2 cell lysate (mock) diluted in 100 μl PBS via intra-tracheal (I.T.) route. Weight change (a) and survival (b) were monitored daily. Data shown are from 5 mice per group. (c) H&E staining of mice lungs infected by HMPV clinical isolates (200x magnification). (d) Pathology scores of the histology slides in (c), evaluated in a group-blinded fashion by a board-certified pathologist. Data shown are from 5 mice per group. (e) Seven-week old BALB/c mice were infected with 2 x 10^5^ PFU live or UV-inactivated HMPV via I.T. route. Fourteen days post-infection (p.i.), bronchoalveolar fluid (BALF) was obtained by injecting 1 ml PBS into mouse lung through cannulated trachea. About 700 μl BALF was recovered from each mouse. Total protein concentration was determined by Bradford assay. Data shown are from 2–3 mice per group. * P<0.05, ** P<0.01, one way ANOVA with Tukey’s multiple comparisons test. (f) Fourteen days p.i., the airway resistance, tissue resistance, and tissue elastance of mice in (e) were analyzed by flexiVent during methacholine challenge. Data shown are from 2–3 mice per group.

We next tested the pathogenesis of the HMPV clinical isolates in an established B6 mouse model for HMPV [26,39]. Comparison of different anesthesia and inoculation routes (intratracheal, I.T. vs. intranasal, I.N.) indicated that I.T. yielded modestly but significantly higher lung viral titer and equivalent nasal titer (**S2 Fig**). We infected each mouse with 1.0 x 10^6^ PFU HMPV or mock LLC-MK2 cell lysate via I.T. route under anesthesia, monitored mice daily for weight loss and survival, and euthanized on day 5 (**S3A Fig**). While mice infected by TN/94-49 and TN/94-66 did not lose weight, mice infected by strains 1501, 1509, C2-202, TN/91-320, and TN/96-35 experienced substantial weight loss and up to 60% mortality (**Fig 1A and 1B**). These mice also showed decreased response to stimuli, hunched posture, huddled behavior, and ruffled fur. Mice infected by strains 42 and C1-334 were moderately ill but recovered.

To exclude the interference of host genetic background, we tested three virulent strains 1501, C2-202, and TN/91-320 together with the avirulent strain TN/94-49 in two other inbred mouse strains BALB/c and DBA/2 (**S3B–S3E Fig**). HMPV TN/94-49 caused no weight loss in either inbred mouse strain, while the three virulent strains induced significant weight loss and mortality in both strains. We previously found DBA/2 mice to be more permissive for HMPV replication than other inbred strains [23,27], and all the DBA/2 mice infected by virulent strains died by 3 days post infection (**S3E Fig**). We confirmed a dose-dependent disease response for C2-202 (**S3F Fig**). UV-inactivated virus did not cause weight loss or death, confirming that disease required viral replication (**S3G Fig**).

### Virulent HMPV clinical isolates cause pulmonary pathology and respiratory dysfunction in mice

To examine pulmonary histology in mice infected by HMPV clinical isolates, we analyzed mouse lungs at 5 days post-infection. The lungs of mock-infected mice showed clear alveoli, but the lungs of mice infected with clinical isolates exhibited various degrees of pathologic changes. The lungs of mice infected with virulent isolates 1501, 1509, C2-202, TN/91-320, and TN/96-35 exhibited more intense H&E staining (**Fig 1C**). Higher magnification examination showed massive neutrophil infiltration and thickening of alveolar walls, characteristic of inflammation and viral pneumonia. Mid-virulent strains 42, TN/94-66, and C1-334 induced mild neutrophil infiltration only surrounding the bronchioles, with clear alveoli. In comparison, TN/94-49 induced minimal pathologic changes (**Fig 1D**). Clinical HMPV isolates also caused airway damage. Total protein concentration was increased in the bronchoalveolar fluid (BALF) of mice infected with C2-202, indicating epithelial damage and leakage of blood proteins into the bronchioles (**Fig 1E**). Mice infected by HMPV virulent isolates 1501 and C2-202 at sub-lethal dose (2 x 10^5^ PFU) survived acute infection but demonstrated altered airway physiology two weeks later (**Fig 1F**). There was a significant difference in airway resistance (Rn) and tissue resistance (G) between TN/94-49 vs C2-202 and C2-202 vs 1501 at 50 mg/ml dose of methacholine. There was a trend toward increased hysteresis as well as C2-202 being higher than the other groups in tissue elastance (H) though this did not reach statistical significance.

### HMPV disease severity does not correlate with lung virus titer

**Fig 2:**
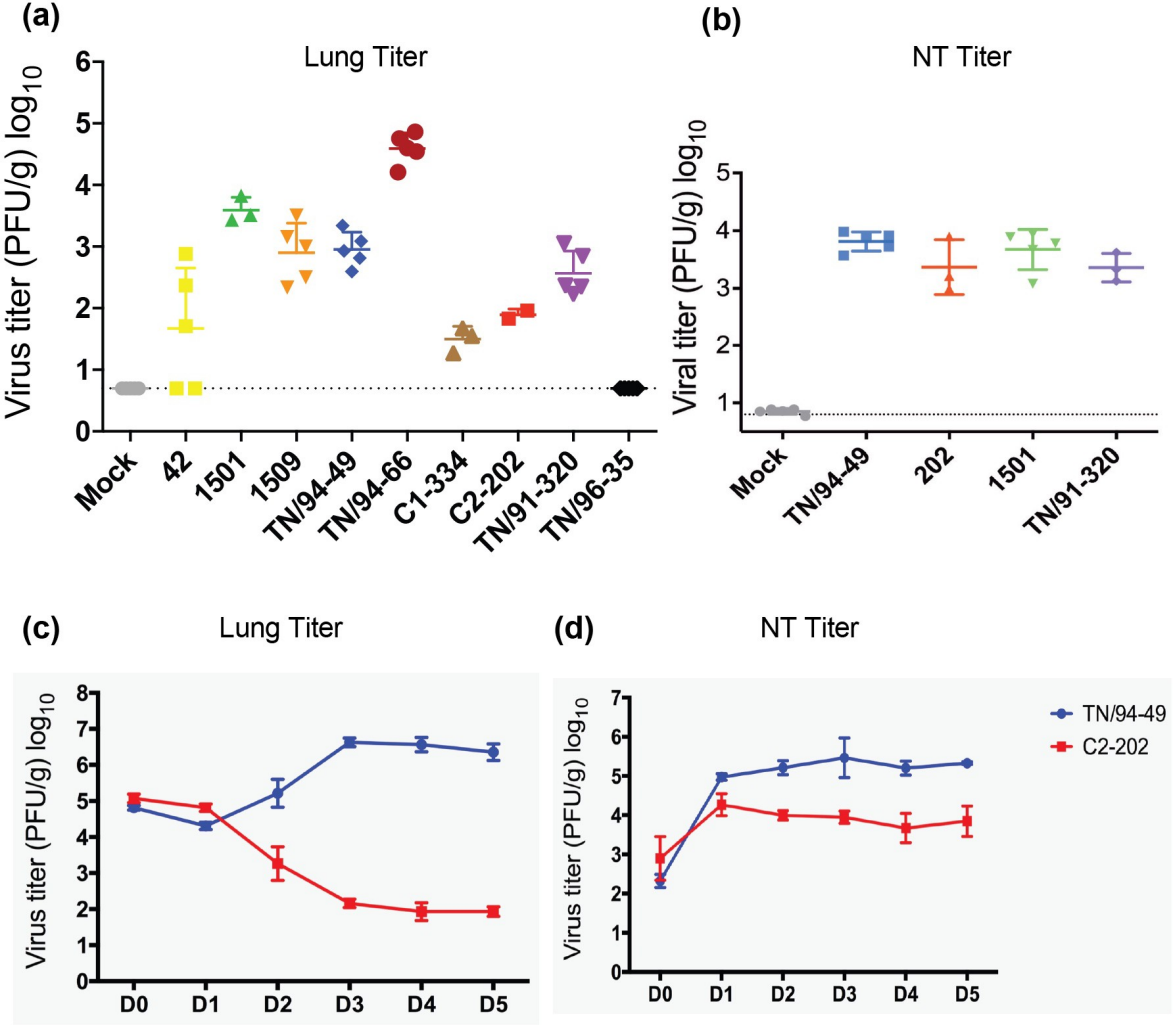
HMPV virulence does not correlate with lung virus titer. (a) Lung and (b) nasal virus titer of HMPV strains in C57BL/6 mice at 5 days p.i. Data are shown as mean ±SD with 5 mice per group except where mice died (C2-202, 2 mice; 1501, 3 mice; C1-334, 3 mice). (c, d) A total of 42 female B6 mice were infected with 1.0 x 10^6^ PFU TN/94-49 or C2-202 via I.T. route. Three mice per group were euthanized per time point and virus titer in lung (c) or nasal turbinate (NT) (d) were determined. Dashed and solid lines represent two experiments on 0–2 days or 2–5 days p.i., respectively. Dotted line on graphs indicates limit of detection. Hpi, hours p.i.

To test the hypothesis that disease severity would correlate with lung virus titer, we measured the lung virus titers of the clinical isolates at 5 days post-infection. The lung virus titers varied widely and did not correlate with virulence of the strain (**Fig 2A**). For example, the non-virulent strain TN/94-66 reached the highest titer (~10^5^ PFU/g). TN/94-49 replicated to 10^3^ in this experiment, but in most other experiments it reached titers of 10^5^−10^6^. Virulent strains ranged from limit of detection (TN/96-35) to ~3.5 x 10^3^ (1501). The nasal turbinate titers were similar among isolates, showing that the inoculations were effective (**Fig 2B**).

To exclude the possibility of different replication kinetics for the clinical isolates, we selected virulent strain C2-202 because it replicated to a high titer in cells and induced the most marked airway dysfunction. We performed a kinetic experiment side-by-side with the non-virulent strain TN/94-49. After a transient eclipse phase on day 1–2, TN/94-49 exhibited log-phase growth, reached peak titer at 3 days p.i., and remained high until 5 days post infection (**Fig 2C**). In contrast, C2-202 exhibited restricted viral growth in the lung (**Fig 2C**), although it replicated to a similar level to TN/94-49 in the nasal turbinates (**Fig 2D**). To further examine the infected lung epithelium, we used flow cytometry to measure HMPV-positive lung epithelial cells. We used a monoclonal anti-HMPV fusion (F) protein antibody (Ab) and EpCAM Ab to identify HMPV F^+^ EpCAM^+^. Mouse lungs infected by C2-202 had fewer HMPV F^+^ EpCAM^+^ lung epithelial cells on day 5 p.i. than TN/94-49 (7.5% 11.2%, respectively) (**S4 Fig**). Collectively, these data suggested that virulent C2-202 lung replication was restricted compared to non-virulent TN/94-49.

### High pro-inflammatory cytokine levels correlate with more severe lung disease

**Fig 3:**
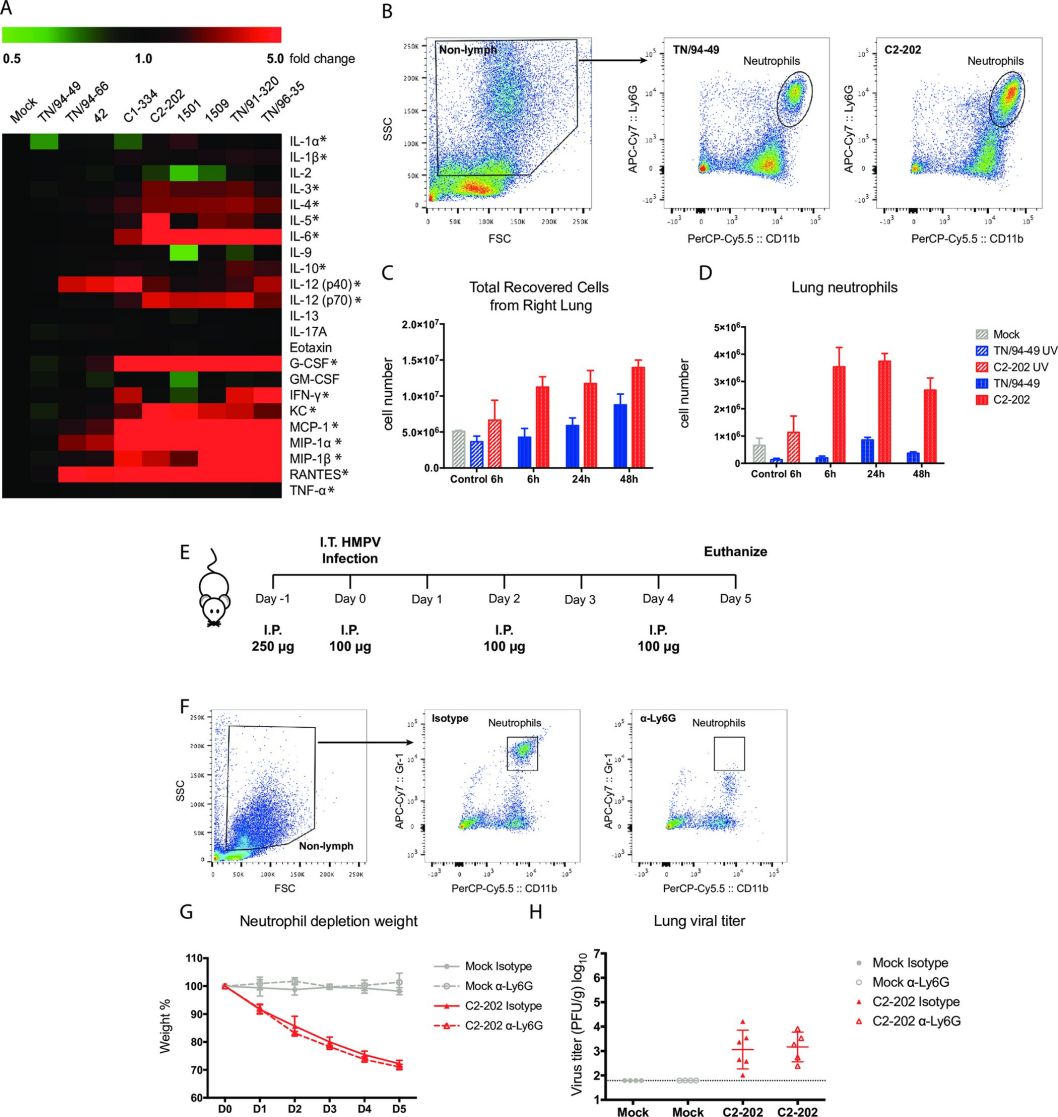
HMPV C2-202 induces pulmonary inflammation and neutrophil infiltration but neutrophil depletion does not rescue disease severity. (A) HMPV pathogenic strains induced higher pro-inflammatory cytokines. C57BL/6 mice were infected with 1.0 x 10^6^ PFU HMPV via I.T. route and lungs harvested 5 days p.i. subject to homogenization. Cytokine protein levels were measured with Bio-Rad mouse 23-plex kit. Absolute cytokine concentration was normalized to that of the mock-infected group (5 mice per group) and average fold changes of each group were plotted in a heat-map with cytokine names on the right and virus strain names on top. More virulent strains are grouped to the right of the graph, and less virulent strains to the left. (B) Gating strategy for lung neutrophils. Neutrophils were gated from F4/80-negative non-lymphocytes as CD11b and Ly6G double positive population. Dead cells were excluded by a violet viability dye before further analysis. (C,D) Kinetics for neutrophil infiltration to lung. Mice were infected by 1.0 x 10^6^ PFU HMPV I.T. and lungs were harvested at the indicated time point for flow cytometry analysis to count total cells (C) or neutrophils (D). (E) Neutrophil depletion does not rescue virulence by HMPV C2-202. Mice were injected via I.P. route with 250 μg α-Ly6G antibody or rat IgG2a isotype antibody 1 day before infection, following with 100 μg antibody I.P on day 0, 2, and 4 post-infection. (F) Depletion was confirmed by flow cytometry. Mice were infected with HMPV I.T. on day 0 as described before. Weight change (G) and day 5 lung virus titer (H) for neutrophil-depleted mice infected with HMPV. Schematic created with BioRender.com.

The limited lung replication of virulent strains and early weight loss kinetics led us to hypothesize that host innate immune response contributed to both disease and viral control. To test this hypothesis, we measured cytokine levels in mouse lungs at 5 days post-infection (**Fig 3A**). More virulent clinical isolates (based on weight loss in Fig 1A, organized to the right of the graph) generally induced a higher level of pro-inflammatory cytokines, including IL-6, IL-12, G-CSF, KC, MCP-1, MIP-1α, MIP-1β, and RANTES compared with the less virulent clinical isolates (**Fig 3A**).

### Pathogenesis of HMPV clinical isolates is inflammasome-independent

The correlation between higher pro-inflammatory cytokine levels and disease severity prompted us to hypothesize that the inflammasome pathway plays an important role in the disease pathogenesis. It has been reported that up-regulation of inflammasome-related genes is correlated with more severe disease symptoms in human patients infected with HMPV [49]. We first examined the inflammasome activation in HMPV-infected cells by immunofluorescence assay. Bone marrow-derived macrophages (BMDMs) were infected with either TN/94-49 or C2-202 at MOI = 1 for 24 hours, fixed, and stained with antibodies recognizing ASC (Apoptosis-associated Speck-like protein containing CARD) and HMPV-F (**S5A Fig**). ASC was evenly distributed in uninfected cells. The formation of inflammasome (as shown by aggregation of ASC) was detected in BMDMs infected by both virus strains, but not in mock-infected or UV-inactivated virus-infected cells (**S5B Fig**). This data shows that HMPV infection is able to activate the inflammasome pathway in virus-infected cells.

We next tested the role of the inflammasome in HMPV disease pathogenesis using genetically-engineered mice lacking inflammasome components ASC or nod-like receptor family pyrin domain containing 3 (NLRP3). We infected age-matched female wild type and ASC-KO mice with 1.0 x 10^6^ PFU HMPV TN/94-49, C2-202, 1501, and TN/91-320. The deficiency in ASC did not reduce the disease severity in the first five days post-infection: ASC-KO mice infected with virulent clinical isolates C2-202, 1501, and TN/91-320 lost about 30% weight, similar to their wild type companions (**S5C Fig**). The more virulent isolates exhibited restricted replication while the less virulent strain TN/94-49 reached about 10^6^ PFU/g in lungs of ASC-KO mice (S3D Fig). We also tested the virulent isolate C2-202 in NLRP3-KO mice. The virulent strain C2-202 was still able to cause about 30% weight loss in NLRP3-KO mice and replicated to low titer, not distinguishable from wild type mice (**S5E and S5F Fig**). Collectively, these data show that NLRP3 and ASC inflammasome are not required for disease pathogenesis of HMPV clinical isolates.

### HMPV virulent isolate C2-202 infection inhibits dendritic cell recruitment

We next used TN/94-49 (non-virulent) and C2-202 (virulent) as two representative strains to characterize the innate immune cell response to HMPV. The antigen presenting cell (APC) recruitment patterns were analyzed with an APC flow panel shown in S6 Fig. We gated for pulmonary dendritic cells (DCs), alveolar macrophages, and interstitial macrophages with surface markers CD11b, CD11c, and MHC-II based on a previous study [39]. To identify APC recruitment patterns, we performed an infection kinetics experiment by harvesting three mice per time point (day 2, 3, 4, and 5 post-infection) and counted the number and percentage of each cell population with flow cytometry. As shown in S7A Fig, HMPV live virus infection induced an increasing number of total cells in lung, compared with mock infection or UV-inactivated virus infection. Although an increasing number of DCs were detected in mice infected by TN/94-49 as infection progressed, the number of DCs in C2-202 infected mice remained low from day 2 to day 5 post infection (**S7B Fig**). However, the amount of MHC-II molecules per cell, as indicated by the mean fluorescent intensity (MFI), did not differ from that of TN/94-49 group (**S7F Fig**). The macrophage recruitment patterns by TN/94-49 and C2-202 infection were also different (**S7C and S7D Fig**). C2-202 induced more alveolar macrophages while TN/94-49 infection led to more interstitial macrophage recruitment. Cells in C2-202 infected lung also expressed higher levels of CD11b (**S7H Fig**), indicating a stronger capacity for infiltration.

In summary, C2-202 infection induced a different APC recruitment pattern compared with TN/94-49, with fewer DC recruitment as the most prominent feature. The role of DCs in C2-202 pathogenesis remains to be assessed.

### HMPV C2-202 induces more neutrophil infiltration but neutrophil depletion does not rescue disease severity

Previously we described neutrophil infiltration into mice lung after HMPV infection by histology staining (Fig 1). We thus sought to further characterize the role of neutrophils in HMPV pathogenesis. To quantify neutrophil infiltration, we used flow cytometry by staining with α-CD11b and α-Ly6G antibodies. Neutrophils were defined as CD11b+ Ly6G+ cells in F4/80-negative non-lymphocyte populations (**Fig 3B**). We infected mice with TN/94-49 and C2-202 via I.T. route and analyzed lung neutrophil numbers at 6h, 24h, and 48h post-infection. We also infected mice with LLC-MK2 cell lysates (mock) or UV-inactivated virus and harvested their lungs at 6h post-infection as controls. HMPV infection induced neutrophil infiltration to lung as early as 6 h post infection and the infiltration peaked during the first day after infection (**Fig 3C and 3D**). Further, HMPV C2-202 induced significantly more neutrophil infiltration to lung compared with TN/94-49 (**Fig 3D**). The higher neutrophil infiltration correlated with the higher KC cytokine level in C2-202 infection (**Fig 3A**).

Based on the correlation between elevated neutrophil infiltration and more severe disease, we hypothesized that neutrophils were the cause for the severe pathogenesis of C2-202 infection. To test this hypothesis, we depleted neutrophils with intra-peritoneal (I.P.) injection of Ab targeting Ly6G (clone 1A8) [50]. This Ab has been used in multiple previous studies to successfully deplete neutrophils in infection models [51–57]. We optimized the Ab dosage by testing different regimens in C57BL/6 mice infected with C2-202 and verified neutrophil depletion efficiency by flow cytometry. After optimization, we performed the neutrophil depletion and infection experiments according to the scheme shown in **Fig 3E**. We confirmed the successful depletion of neutrophils by flow cytometry staining (**Fig 3F**). However, neutrophil depletion did not rescue the severe pathogenesis of C2-202 as the mice without neutrophils still lost about 30% weight at 5 days post infection (**Fig 3G**) and the absence of neutrophils did not affect virus replication (**Fig 3H**).

### HMPV C2-202 infection induces higher type I and III IFN

**Fig 4:**
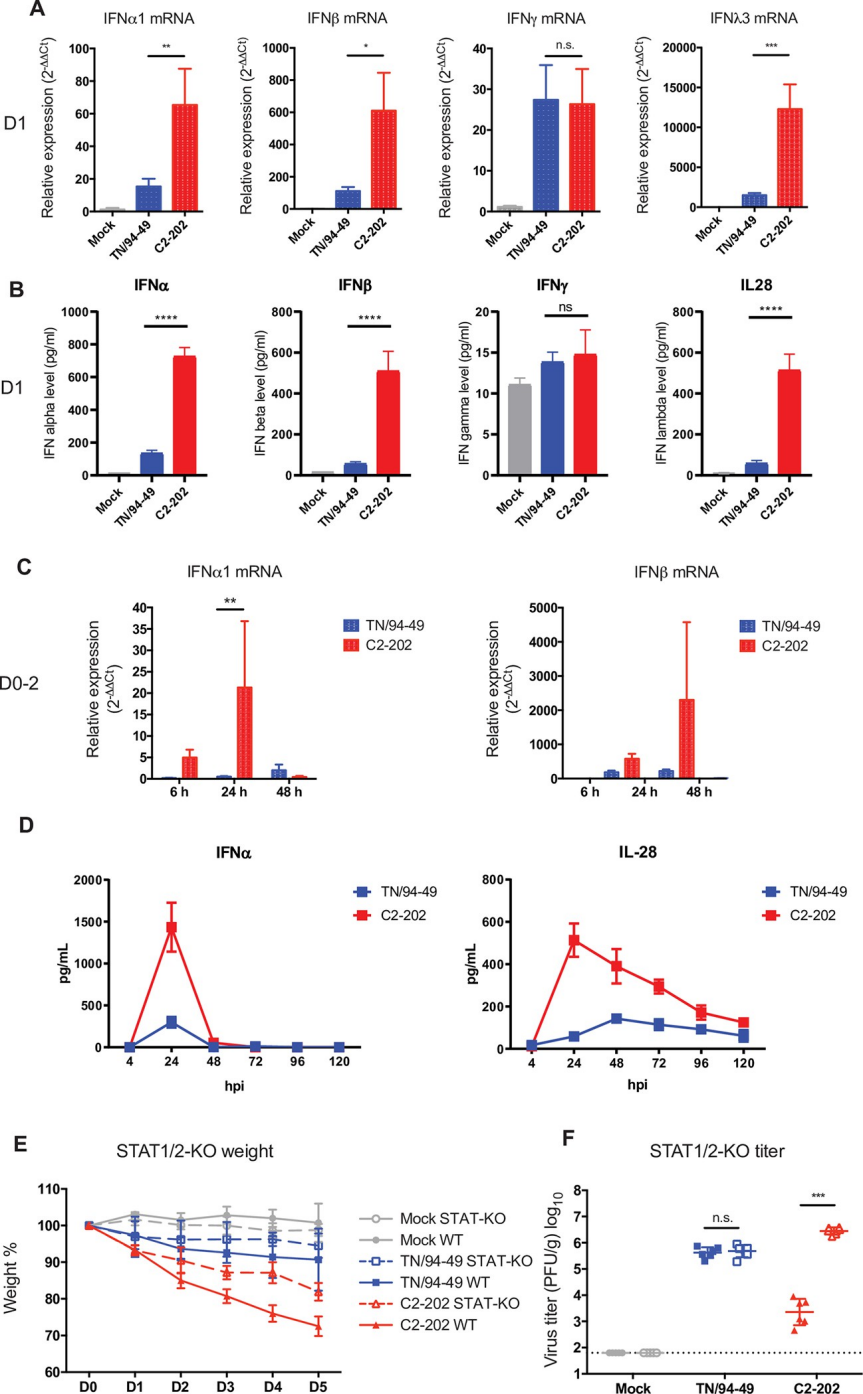
Type I and III IFN are induced by C2-202 and deficiency in IFN signaling reduces disease severity. (A) Relative expression of IFNα1, IFNβ, IFNγ, and IFNλ3 mRNA in mouse lung at 1 day post-infection. Relative expression level was obtained by qRT-PCR and normalized to HPRT expression. Fold changes normalized to mock infection are shown. (B) Protein level of IFNα1, IFNβ, IFNγ, and IL-28 measured by ELISA. (C) Relative expression of IFNα1 and IFNβ mRNA in mouse lung at 6, 24, and 48h post-infection. Relative expression level was obtained by qRT-PCR and normalized to HPRT. Fold changes normalized to mock are shown. * P<0.05, ** P<0.01, *** P<0.001, n.s. P>0.05 by student’s t-test. (D) Protein level of IFNα1 and IL-28 measured by ELISA at multiple time points post-infection. (E) Weight change and (F) day 5 lung virus titer of wild type (WT) or STAT1/2-deficient mice (STAT-KO) infected with 5.0 x 10^5^ PFU mock, TN/94-49, or C2-202. Solid lines with closed shapes are wild type mice and dashed lines with open shapes are STAT1/2-deficient mice. Data were pooled from a total of 5–6 mice per group from two independent experiments. *** P<0.001, n.s. P>0.05 by student’s t-test.

IFNs are key regulators of the host immune response against viral infection. Type I and III IFN mediate the innate anti-viral and pro-inflammatory responses, and type II mediates the adaptive response. We measured the IFN induction levels in the mouse lungs by extracting total RNA from homogenized lung tissue at 1 day post-infection, and performed qRT-PCR to detect IFNα1, IFNβ, IFNγ, and IFNλ3 expression levels. HMPV infection induced all three types of IFN at 1 day post infection. Interestingly, C2-202 induced a much higher type I (IFNα1, IFNβ) and type III (IFNλ3), but not type II (IFNγ) response compared with TN/94-49 infection (**Fig 4A**). Measurement of IFN protein level by ELISA confirmed these findings (**Fig 4B**). IFNα1 mRNA peaked 24h post-infection, while IFNβ was elevated 48h post-infection (**Fig 4C**). The IFN response was tightly regulated, as type I IFN protein levels dramatically decreased on day 2 post-infection after a transient peak on day 1, while IL-28 exhibited a similar peak on day 1 post-infection and slower decline (**Fig 4D**).

### Deficiency in IFN signaling reduces disease severity

To further characterize the role of type I and type III IFN in the pathogenesis of HMPV clinical isolates, we infected genetically engineered STAT1/2-deficient (STAT-KO) mice, which are deficient in both type I and III IFN signaling due to the absence of signal transducers. Deficiency in STAT1/2 partially rescued the C2-202 disease phenotype (**Fig 4E**). The STAT-KO mice infected by C2-202 experienced less weight loss and were more active than their wild type counterparts. The deficiency in IFN signaling also restored the replication ability of C2-202 in lung. HMPV C2-202 was able to replicate 1000-fold better in STAT-KO mice than in wild type mice, and the titer was similar to that of TN/94-49 (about 10^6^ PFU/g) (**Fig 4F**). HMPV TN/94-49 replicated similarly in STAT-KO mice and wild type mice. Downstream cytokines including IL-6, MCP-1, MIP-1A, MIP-1B, and RANTES induction by C2-202 were lower in IFNAR KO and STAT-KO mice compared to WT (**S9 Fig**). Collectively, these data suggest that type I and III IFN signaling control HMPV replication and induce immunopathology.

### Deficiency in type I IFN signaling alone rescues disease of HMPV C2-202

**Fig 5:**
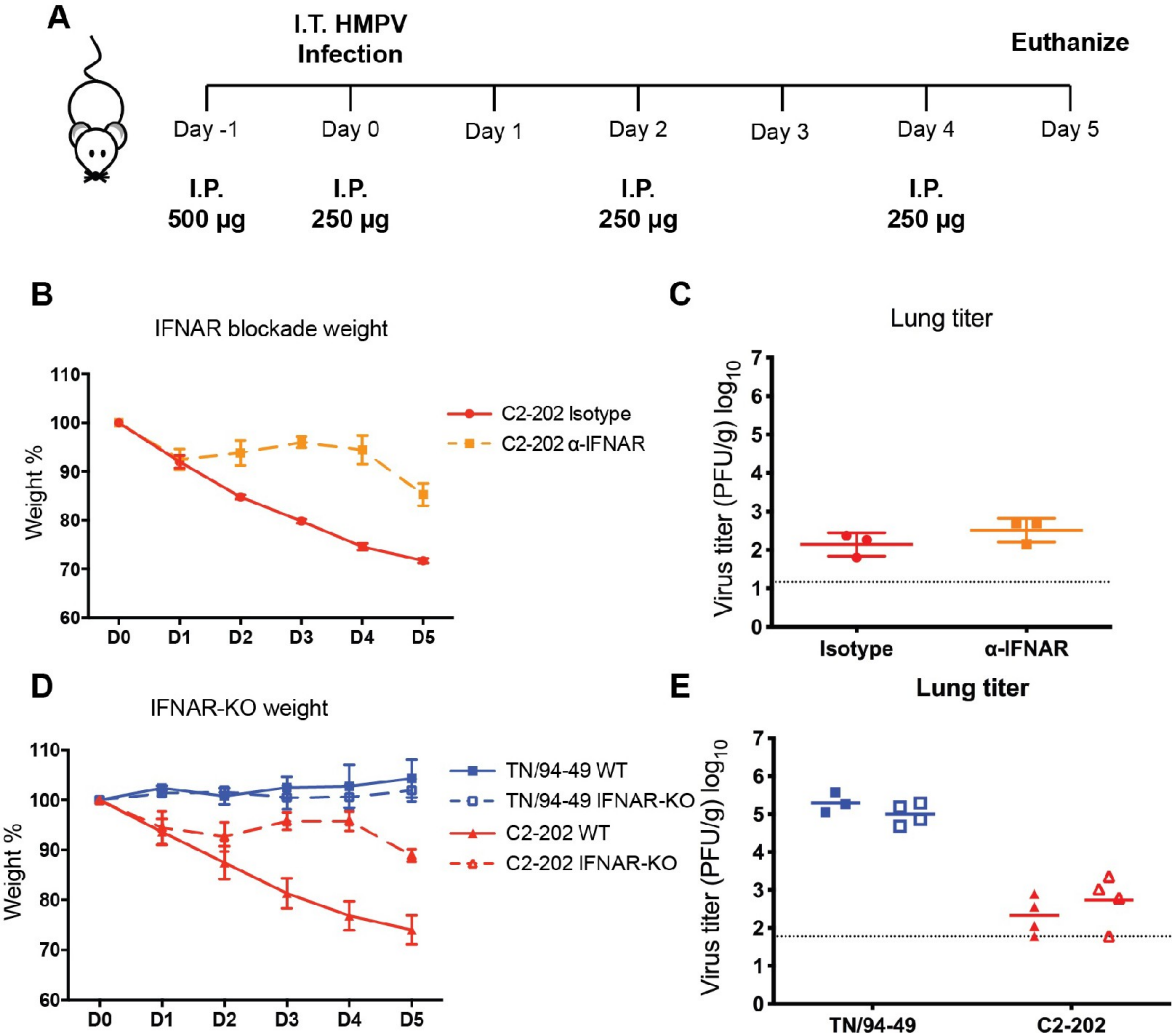
Deficiency in type I IFN signaling alone rescues disease of HMPV C2-202. (A) Experimental design for IFNAR blockade. (B) Weight change and (C) day 5 lung virus titer of mice injected with isotype or IFNAR blockade antibody and infected with 5.0 x 10^5^ PFU C2-202. (D) Weight change and (E) day 5 lung virus titer of wild type (WT) and IFNAR-deficient (IFNAR-KO) mice infected with 5.0 x 10^5^ PFU C2-202. Data are shown as mean ±SD from three to four mice per group. Schematic created with BioRender.com.

We next tested whether type I IFN alone was sufficient to induce anti-viral response and immunopathology using two mouse models that were deficient in type I but competent in type III IFN signaling. In the IFN-α/β receptor (IFNAR) blockade model, we treated the mice with IFNAR blocking Ab or isotype Ab for four doses before and during C2-202 infection (**Fig 5A**). The IFNAR blockade treatment significantly rescued the acute disease phenotype (**Fig 5B**). However, there was no change in the restriction of viral replication (**Fig 5C**). We also tested this hypothesis in mice genetically deficient in IFNAR (IFNAR-KO). Similar phenotypes were observed in the IFNAR-KO mice, with substantial rescue of disease phenotype but no effect on viral replication (**Fig 5D and 5E**). As previously shown, TN/94-49 replication was not restricted in WT or IFNAR-KO mice. Collectively, these data show that type I IFN signaling pathway mediated the immuno-pathogenesis of HMPV C2-202 infection but had little effect on controlling viral replication.

### Type III IFN contributes to virus control but not disease

**Fig 6:**
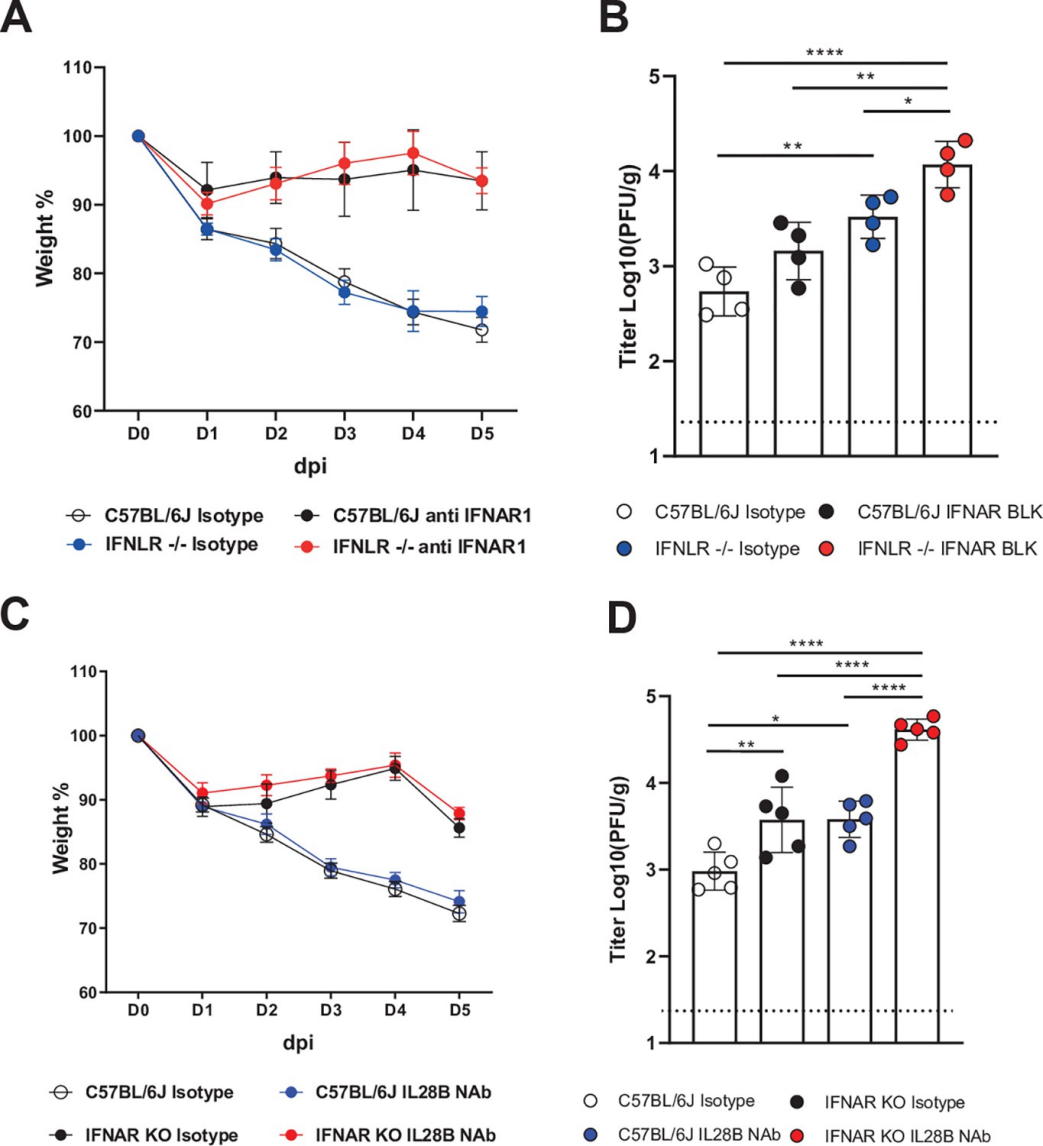
Type III IFN contributes to virus control but not disease. (A) Weight change and (B) day 5 lung virus titer of WT and IFNLR^-/-^ mice injected with isotype Ab or IFNAR neutralizing Ab and infected with 5.0 x 10^5^ PFU C2-202. (C) Weight change and (D) day 5 lung virus titer of wild type (WT) and IFNAR-deficient (IFNAR-KO) mice injected with isotype Ab or IFN-λ (IL28B) neutralizing Ab and infected with 5.0 x 10^5^ PFU C2-202. Data are shown as mean ±SD from five mice per group. * P<0.05, ** P<0.01, *** P<0.005, ****p<0.001, one way ANOVA with Tukey’s multiple comparisons test.

To dissect the individual roles of type I and III IFN, we used mouse models that we depleted either type I or type III or both. Wildtype C57BL/6J or interferon λ receptor knockout mouse (*Ifnlr*^-/-^) were treated with isotype control or IFNAR blocking Ab on day 1 prior to infection with 5x10^5^ PFU of virulent strain C2-202 and on day 1 and 3 post-infection. WT B6 and *Ifnlr*^-/-^ mice treated with isotype control lost >25% of body weight over 5 days, while WT C57BL/6J and *Ifnlr*^-/-^ mice treated with IFNAR antibody experienced minimal weight loss (**Fig 6A**). Next, we evaluated how type I and III IFN affected lung viral titer. *Ifnlr*^-/-^ mice treated with isotype Ab showed a modest but significant 0.8 log_10_ increase in lung virus compared to WT B6 treated with isotype Ab (**Fig 6B**). In comparison, blocking IFNAR alone in B6 showed only 0.4 log_10_ on viral shedding, but IFNAR blockade in *Ifnlr*^-/-^ mice restored viral titer to 4.1 log_10_ (**Fig 6B**). In a separate experiment, we infected WT, IFNAR-KO, and *Ifnlr*^-/-^ mice and collected lungs for titer at different time points to 14 days (**S10 Fig**). WT and *Ifnlr*^-/-^ mice lost weight, while IFNAR-KO mice did not. *Ifnlr*^-/-^ mice had higher viral titers on day 5, while all animals cleared by day 10.

Alternatively, we selectively blocked type I IFN or type III IFN or both by using IFNAR-KO mice and IFN-l (IL28B) neutralizing Ab. WT or IFNAR-KO mice were treated with isotype control or IL-28B neutralizing Ab on day 1 prior to infection with 5x10^5^ PFU of virulent C2-202 and on days 1 and 3 post-infection. WT mice treated with either isotype control or IL28B neutralizing Ab lost >25% of body weight over 5 days, while IFNAR-KO mice treated with isotype or IL28B neutralizing Ab experienced minimal weight loss (**Fig 6C**). However, blockade of IL28B in IFNAR-KO mice restored lung viral replication of C2-202 to ~10^5^ PFU/g compared with IFNAR-KO mice treated with isotype Ab, a 1.6 log_10_ increase in viral replication, while there was modest change in viral titer in WT mice treated with isotype or IL28B neutralizing Ab (**Fig 6D**). Collectively, these results suggest that IFN-I contributes primarily to disease, while IFN-III principally functions to control viral replication.

## Discussion

Our study reveals two important aspects of HMPV pathogenesis. First, different clinical isolates exhibit markedly differing virulence and distinct inflammatory responses. Moreover, type I and type III IFN exhibit distinct functions in response to HMPV. Many viruses exhibit quite disparate biology, virulence, or even receptor usage between cell-adapted laboratory/vaccine strains and clinical isolates, including RSV [30,31,58]; cytomegalovirus, herpes simplex, and varicella virus [59–61]; Dengue virus [62]; and measles virus [63,64]. Indeed, a classic method of viral attenuation to generate a vaccine candidate is cell passage. We found a spectrum of *in vivo* phenotypes between clinical isolates and several lab-adapted strains; moreover, virulence did not correlate with genetic subgroup and importantly was confirmed in multiple inbred mouse strains. Clinical studies have sought but not consistently shown differences in human disease between subgroups [65–69]. These data will provide powerful tools to explore mechanisms of HMPV pathogenesis and help elucidate the contribution of specific viral proteins.

Clinical disease and inflammation were not correlated with lung viral titer, consistent with immunopathology caused by both HMPV and RSV [39,70–74]. Virulent HMPV strains were associated with markedly increased proinflammatory cytokine production and neutrophil influx. However, experiments in genetically deficient mice and neutrophil depletion demonstrated that these were not required for pathogenesis. Although neutrophil depletion did not alter disease in these experiments, neutrophils express *Ifnlr*; future experiments using bone marrow chimeras could confirm whether immune or non-immune cells expressing *Ifnlr* are responsible for virus control. A recent paper reported that chemical inhibition of NLRP3 reduced disease [75]; however, the effect was more marked in BALB/c mice, which may have more severe disease with some strains of HMPV [25,71]. Similarly, depletion of neutrophils enhanced disease in BALB/c mice [51]. These differences could be due to different virus strains; the panel of genetically and phenotypically distinct isolates collected herein may enable elucidation of specific viral protein-cell protein interactions that drive these outcomes.

The virulent isolate C2-202 and the avirulent TN/94-49 induced markedly different macrophage and dendritic cell responses, which may contribute to the pro-inflammatory phenotype. The effects of the differential response of antigen-presenting cells warrants future studies of adaptive immune response to different HMPV strains; dendritic cells are particularly important for T cell response to HMPV [76]. Virulent C2-202 induced potent production of type I and III IFN, and inhibition of type I IFN signaling reduced pathology with minimal effect on viral replication. Modulation of type I IFN activation by receptor antibody blockade or depletion of key mediators of IFN signaling alleviates disease severity of RSV, influenza virus [77], lymphocytic choriomeningitis virus (LCMV) [78,79], and others. We previously showed that type I IFN signaling controls early HMPV replication but leads to airway dysfunction and inflammation in HMPV infection [39]. Here, we demonstrate that type I IFN primarily contributes to disease phenotype.

We found that type III IFN signaling had no effect on pathogenesis but inhibited viral replication. One previous study reported that HMPV induced type III IFN and that treatment of mice with exogenous type III IFN reduced viral titer, but also ameliorated weight loss; however, that study used BALB/c mice [40]. Type I IFN have been reported to contribute to antiviral immunity more potently during systemic infections, while type III exhibits a greater role in mucosal surface infections [80,81]. HMPV infects airway epithelial cells and does not infect other organs [21,23,82]. Thus, modulation of type I or type III IFN might represent a therapeutic intervention against HMPV.

Collectively, these results demonstrate that diverse HMPV clinical isolates exhibit widely divergent virulence *in vivo* using a mouse model. Moreover, the findings suggest discordant roles for type I and type III IFN. While pathogenesis in mice may differ from that in humans, the use of these clinical isolates will facilitate efforts to dissect viral and host determinants of HMPV virulence.

## Materials and methods

### Ethics statement

The research was approved by the University of Pittsburgh Institutional Animal Care and Use Committee #21028897.

### Optimization of infection route for HMPV

Since mice are not natural hosts for HMPV, we first optimized infection routes and anesthesia methods to establish an effective infection procedure for C57BL/6 (B6) mice. We compared the virus yield from intranasal (I.N.) and intratracheal (I.T.) routes. We also compared isoflurane to ketamine/xylazine for anesthesia. Higher lung virus titer was obtained using the intratracheal (I.T) route compared to the intranasal (I.N.) route (**S2 Fig**). All the subsequent mouse studies used this I.T. route with isoflurane anesthesia. The intratracheal route has been widely used in drug delivery for local treatment of rhinitis or nasal polyposis, as well as in influenza pathogenesis studies. Under these conditions, most of the inoculum is aspirated into the lungs [83–86].

### Cells and viruses

LLC-MK2 (ATCC CCL-7) cells were maintained in OPTI-MEM medium containing 2% heat-inactivated fetal bovine serum (FBS). The HMPV clinical isolates we tested were obtained through the Vanderbilt Vaccine Clinic and Cincinnati Children’s Hospital from 1999 to 2004[2,3,7]. Specimens positive for HMPV by RT-PCR were passaged in LLC-MK2 cells for 5 to 10 passages and confirmed by immunofluorescence. The cell cultures also tested negative for human RSV, parainfluenza viruses 1–4, human rhinovirus, and influenza by RT-PCR. Master stocks of laboratory adapted clinical strain TN/94-49 and clinical isolates were generated. Working stocks of HMPV were purified through a 20% sucrose cushion in ultracentrifuge at 120,000 ×g for 2 hours. Primary bone marrow-derived macrophages (BMDMs) and bone marrow-derived DCs (BMDCs) were generated as previously described [26]. In brief, bone marrow progenitor cells were obtained from B6 mice and resuspended in R10 medium (RPMI 1640 [Corning] plus 10% FBS, 2 mM glutamine, 50 μg/ml gentamicin, 2.5 μg/ml amphotericin B, and 50 μM β-mercaptoethanol [Gibco, Invitrogen]). For BMDM, cells were cultured in R10 supplemented with 20 ng/μl rmM-CSF (Peprotech). Fresh medium with rM-CSF was replenished on days 2 and 4. Attached cells on day 6 were used for experiments. For BMDC, bone marrow progenitor cells were cultured in R10 supplemented with 20 ng/μl rmGM-CSF and 10 ng/μl rmIL-4 (Peprotech). 75% of medium was replaced on day 3, and at day 6 cells were counted and plated for experiments. HMPV laboratory adapted clinical strain TN/94-49 (genotype A2), was grown and titered in LLC-MK2 cells as described previously [87].

### Animals

C57BL/6J, C57BL/6T, BALB/cJ, DBA/2J mice were purchased from commercial breeders (Jackson Laboratory or Taconic), and ASC-deficient (ASC^-/-^) B6 mice were kindly provided by Dr. John Alcorn. Nlrp3-deficient (Nlrp3^-/-^) B6 mice were kindly provided by Dr. Jieru Wang. IFNAR IFN-α/β receptor-deficient (IFNAR^-/-^) B6 mice were kindly provided by Dr. Terence Dermody. All animals were bred and maintained under specific-pathogen-free conditions in accordance with the guidelines of the University of Pittsburgh Institutional Animal Care and Use Committee. *Ifnlr*^-/-^ mice were generated and backcrossed on a B6 background by the University of Pittsburgh Transgenic Mouse Core. Age- and gender-matched animals were used in all experiments. Mice from different backgrounds were co-housed for two weeks before any experiment.

For all experiments, mice were anesthetized by intraperitoneal injection of ketamine-xylazine or by inhalation isoflurane followed by intranasal or intratracheal administration of sucrose purified HMPV as indicated. After infection, the animals were evaluated daily for weight loss, mortality and symptoms. Animals were euthanized at indicated times postinfection. Nasal turbinates and right lungs were collected for virus titration. For histopathology analysis, a portion of the left lung was inflated with 10% buffered formalin, paraffin embedded, stained, and analyzed using a formal scoring system in a group-blinded fashion by an experienced lung pathologist as previously described. The studies were approved by the University of Pittsburgh Institutional Animal Care and Use Committee #18032376. Animals were euthanized by CO_2_ asphyxiation.

### Neutrophil depletion

To deplete neutrophils, 6- to 12-week-old B6 mice were given 100μg anti-Ly6G (clone 1A8, BioXCell) injected intraperitoneally (i.p.) 1 day prior to infection, and again on days 1 and 3 postinfection. Control mice were treated with rat IgG1 isotype control (2A3; Bio X Cell) on day −1, 1, and 3 postinfection. The efficiency of the blockade was confirmed by flowcytometry using mouse Ly-6G/Ly-6C (Gr-1, clone RB6-8C5) Ab.

### IFN-α receptor blockade

C57BL/6J or *Ifnlr*^-/-^ mice were injected i.p. with 500μg anti mouse IFNAR-1 Ab (clone MAR1-5A3, BioXCell) or isotype control mouse IgG1 in 0.1 ml of PBS 1 day prior to infection or on indicated time post-infection, followed by 250 μg Ab or isotype control every other day.

### IFN-λ IFN depletion

To deplete IFN type III IFN, 6 to 12-week-old C57BL/6J or IFNAR KO mice were given 300ug of murine IL28B neutralizing Ab (clone 3C11, InvivoGen) intraperitoneally (i.p.) 1 day prior to infection, and subsequently given 150ug on days 1, and 3 postinfection. Control mice were treated with mice IgG1 isotype control (clone MOPC-21; Bio X Cell) on days −1, 1, and 3 postinfection. Mice were monitored daily for weight change and signs of disease. On Day 5 post infection, mice were euthanized and lung viral titer was determined by plaque assay.

### Flow cytometry

Animals were euthanized at indicated times post-infection. The left lung was collected for virus titration and the right lungs were processed into a single cell suspension as previously described [26]. Briefly, lungs were rinsed in R10 medium, minced with a scalpel, and then incubated with 2 mg/ml collagenase A (Roche) and 20 μg/ml DNase (Roche) for 1 h at 37°C. Single-cell suspensions of digested lungs were obtained by pressing lung tissue through a nylon cell strainer (70-μm pore size). Erythrocytes were lysed with red blood cell lysis buffer (Sigma). Surface staining was performed for 1 h at room temperature in phosphate-buffered saline (PBS) containing 2% FBS. For innate immune cell characterization, lung cells were stained with anti-epithelial cell adhesion molecule (EpCAM, clone G8.8; BioLegend), anti-CD11c (clone HL3; BD), anti-CD11b (clone M1/70; Tonbo), anti-major histocompatibility complex (MHC) class II (clone 2G9; eBioscience), anti-Gr-1 (clone RB6-8C5; Biolegend), and anti-Ly6G (clone 1A8; BioLegend) antibodies. Cells were also stained with human anti-HMPV-F Ab 54G10 to detect HMPV antigens. For all cell populations, forward scatter (FSC) and side scatter (SSC) gating was used to obtain cells of the appropriate size and shape and doublets excluded. A Pacific blue Live/Dead staining dye was used to exclude all dead cells. Flow cytometric data were collected using an LSR II (BD Biosciences) and analyzed with FlowJo (Tree Star).

### Next generation sequencing of virus genome and sequence analysis

Total RNA was extracted from sucrose-purified HMPV virus stocks using Trizol reagent (Invitrogen). The RNA samples for all HMPV clinical isolates were subjected to ribosomal RNA depletion, fragmentation, library preparation, and sequenced on a 318 chip with an Ion PGM system (ThermoFisher) at the University of Pittsburgh Genomics Research Core. Sequencing reads were assembled and aligned to a reference in CLC Genomics Workbench (QIAGEN) to generate a consensus genome sequence. Genome sequence for HMPV TN/94-49 (JN184400) and NL/1-99 (AY525843) were used as the reference sequence for subgroup A and B, respectively. Phylogenic trees were generated by aligning nucleic acid sequences using the ClustalW algorithm in MacVector 14 (MacVector) [88] and the neighbor-joining method.

### Multistep virus growth curves in LLC-MK2 cells

Confluent LLC-MK2 cells in T25 flasks were infected with HMPV clinical isolates at MOI of 0.1 in triplicates. After 1 h of adsorption at 37°C, the inoculum was removed and the cells were washed three times with PBS. Fresh Opti-MEM containing 0.5μg/ml trypsin-TPCK was added, and the infected cells were incubated at 37°C in a CO_2_ incubator. At indicated time points post-infection, 200 μl supernatant was collected from each well, snap-frozen, and stored at -80C. The virus titer was determined by an immunostaining assay in LLC-MK2 cells as described above.

### Pulmonary cytokine/chemokine levels

Mice lung homogenates or BAL fluid were clarified by centrifugation at 13,800 × g for 10 min at 4°C, and 50uL of the supernatant was used. Cytokines and chemokines from clarified BAL fluid or lung homogenate were measured using Bio-Plex Mouse Cytokine 23-Plex (BioRad) or ELISA (R&D) according to manufacturer’s recommendations.

### Real-time PCR

Total RNA from 200-μl volume of undiluted lung homogenate from infected or uninfected mice was extracted using RNeasy kit according to manufacturer’s recommendations (Qiagen) and stored at −80°C. Real-time reverse transcription (RT)-PCR was performed with 25-μl reaction mixtures containing 5 μl of extracted RNA on an ABI StepOnePlus real-time PCR system (Life Technologies/Applied Biosystems) with the AgPath-ID One-Step RT-PCR kit (Life Technologies/Applied Biosystems). For IFN α1, IFN β, and hypoxanthine phosphoribosyltransferase (HPRT) gene expression, primers and probes were used according to the manufacturer’s instructions (Applied Biosystems/Ambion). All values were normalized to the HPRT expression. Gene expression are reported as fold differences between mock infected and infected by the ΔΔCT method.

### Lung mechanics measurements

Following experimental exposures, pulmonary function was assessed by mechanical ventilation of anesthetized (90 mg/kg pentobarbital-NA, i.p.) and tracheotomized mice using a computer-controlled small-animal mechanical ventilator (flexiVent; SCIREQ) as previously described [88,89]. Mice were mechanically ventilated at 200 breaths/min with a tidal volume of 0.25 ml and a positive end-expiratory pressure of 3 cm H2O (mimicking spontaneous ventilation). Respiratory mechanics measurements were made prior to and following the administration of the drug methacholine (dose range: 0–50 mg/ml), which causes the smooth muscle surrounding the airways to constrict. Multiple linear regression was used to fit measured pressure and volume in each individual mouse to the linear model of the lung. Model fits that resulted in a coefficient of determination < 0.8 were excluded.

### Statistical analysis

Data analysis was performed using Prism version 6 (GraphPad Software). Multiple group comparisons were performed using two-way ANOVA followed by Turkey post-test for individual group comparisons. Error bars in each graph represent SD.

## Supporting information

S1 Fig Phylogenetic and growth characteristics of HMPV clinical isolates. (a-c) Phylogenic trees for nine HMPV clinical isolate strains together with reference strain NL/1/00 (AF371337), NL/1/99 (AY525843), and CAN/97-83 (AY297749) were generated by MacVector 16 software based on the nucleotide sequence of the identified full-length N (a), F (b), or G (c) genes. The ClustalW algorithm was used to align the sequences before creating phylogenic trees with neighbor joining method. Branch lengths are proportional to the inferred amount of nucleotide changes. (d) Growth kinetics of HMPV clinical isolate strains 1501 (A1), TN/94-49 (A2), C2-202 (B1), and TN/91-320 (B2) in LLC-MK2 cells. Cells were inoculated with MOI = 0.1 of each isolate and incubated with growth medium containing 0.5 μg/ml trypsin. Each day, 200 μl of supernatant fluid was collected and snap-frozen, and fresh medium was supplemented. Data were shown as mean with SD from three replicates. I Representative images of HMPV clinical isolates infected LLC-MK2 cells at 5 days post infection. Scale bar 200 μm.
(TIF)

S2 Fig Intratracheal (I.T) inoculation yields slightly higher lung titer than intranasal (I.N.) inoculation. Seven-week-old female B6 mice were anesthezed with inhaled isoflurane (Iso) or injected ketamine/xylazine (Keta, Xyla) and inoculated with 1.0 x 10^6^ PFU HMPV TN/94-49 in 100 μl via I.T. on I.N. route. Weight (a) was monitored daily, and lung (b), and nasal turbinate (c) viral titer determined on D5. * = P<0.05, one-way ANOVA.
(TIF)

S3 Fig HMPV clinical isolates cause differential weight loss and mortality in mouse modea) Design for animal experiments. (b,c) Seven-week old female BALB/c mice were infected with 1.0 x 10^6^ PFU HMPV clinical isolates or comparable amount of LLC-MK2 lysates (mock) diluted in 100 μl PBS via I.T. route. Weight loss (b) and survival (c) were monitored daily. N = 5 mice per group. Similar experiments were performed in age-matched female DBA/2 mice (d,e) with 5 mice per group. (f) Dose-dependent weight loss by HMPV clinical isolate C2-202. C57BL/6 mice were infected by varying dosages of C2-202 or 1.0 x 10^6^ PFU TN/94-49 diluted in 100 μl PBS via I.T. route. Data are shown from 5 mice per group except where mice died, leaving fewer. (g) C57BL/6 mice were infected by 1.0 x 106 PFU live or UV-inactivated TN/94-49 and C2-202 via I.T. route. Data are shown from 2–6 mice per group. Schematic created with BioRender.com.
(TIF)

S4 Fig Lung epithelial cells infected by C2-202 or TN/94-49 on day 5. Mice were infected as described and on day 5 post-infection, a single-sell suspension was prepared from lung homogenate. Cells were stained with a monoclonal anti-HMPV F mAb 54G10 and anti-EpCAM Ab. Cells were analyzed by flow cytometry gated by forward and side scatter, and HMPV F^+^ EpCAM^+^. Mouse lungs infected by C2-202 had 7.5% HMPV F^+^ EpCAM^+^ lung epithelial cells on day 5 p.i. vs. TN/94-49 (11.2%). Representative plots from replicate experiments is shown.
(TIF)

S5 Fig The inflammasome pathway is activated in HMPV-infected bone marrow-derived macrophages but does not correlate with disease severity. (a) Culture scheme of murine bone marrow-derived macrophages (BMDMs). (b) Immunofluorescent assay on HMPV-infected, mock-infected, or UV-inactivated HMPV infected BMDMs. ASC was labeled with fluorophore AF568 (red), and HMPV-F was labeled with fluorophore AF488 (green). The nucleus was stained by DAPI (blue). The white arrowhead points to the aggregated ASC indicating formation of inflammasome structure. Scale bar 100 μm. (c,d) Weight change and lung titer of HMPV clinical isolates in ASC-KO mice. Female wild type C57BL/6 or ASC-KO mice were infected with 1.0 x 10^6^ PFU HMPV clinical isolates via I.T route. The lungs were harvested 5 days post-infection for titration. Data are shown as mean ±SD from three mice per group. (e,f) Weight change and lung titer of TN/94-49 and C2-202 in NLRP3-KO mice. Age-matched male and female wild type C57BL/6 or NLRP3-KO mice were infected with 1.0 x 10^6^ PFU TN/94-49 or C2-202 via I.T route. The lungs were harvested 5 days post-infection for titration. Data are shown as mean ±SD from 2–3 mice per group. All knock-out mice were co-housed with WT for 2 weeks before experiments. Schematic created with BioRender.com.
(PNG)

S6 Fig Gating strategy for mouse lung antigen presenting cells. Single cell suspension from lungs of C57BL/6 mice were prepared as described in Methods. The lung cells were treated with Fc-block reagent before staining with surface markers CD11b, CD11c, and MHC-II. Flow cytometric data were analyzed with FlowJo software. The panel shown was gated on single live cells after excluding doublets by forward and aide scatter.
(TIF)

S7 Fig HMPV virulent isolate C2-202 infection inhibits dendritic cell recruitment. Quantification of flow cytometry analysis on inflammatory cell recruitment following HMPV TN/94-49 and C2-202 infection in a time-course experiment. The number of total recovered cells (a), dendritic cells (b), alveolar macrophages (c), and interstitial macrophages (d) for each time point were shown as mean ±SD from three mice per group. The control group sample were collected at day 5 post infection. The percentage of marker-positive cells in total cells and mean fluorescent intensity (MFI) of MHC-II (e,f) and CD11b (g,h) were also shown as mean ±SD as above. * P<0.05, ** P<0.01, *** P<0.005, ****p<0.001, ns p>0.05, t-test with correction for multiple comparisons.
(TIF)

S8 Fig Virulent C2-202 caused greater histopathology. WT B6 mice were infected with 2.5 x 10^5^ of either C2-202 or TN/94-49. Histopathological scores show that the pathogenic isolates C2-202 induced greater histopathological changes on days 7 and 10, with a slight decrease on day 14. * P<0.05, ** P<0.01 by student’s t-test.
(TIF)

S9 Fig IFN signaling is required for maximum cytokine production. WT B6, IFNAR KO, and STAT-KO mice were infected with 2.5 x 10^5^ of C2-202, euthanized on day 5, and lung homogenate cytokines were measured by ELISA.
(TIF)

S10 Fig IFNAR signaling contributes to disease. WT, IFNAR KO, and *Ifnlr*^-/-^ (IFNLR KO) mice were infected with 2.5 x 10^5^ of C2-202 and weighed daily. Subgroups of mice were euthanized on the indicated day post-infection to measure lung virus titer.
(TIF)

S1 Table The coding sequences of nine HMPV proteins of the nine clinical isolates were aligned in MacVector 16 software with ClustalW algorithm. The mean, maximum, and minimum sequence identity (%) of each paired comparison between any two isolates were listed in the table. Abbreviations: nucleotide (nt), amino acid (aa), maximum identity (max), and minimum identity (min).
(DOCX)
