# Supplementary figures and images for: Distinct roles for type I and type III interferons in virulent human metapneumovirus pathogenesis
Source: PLoS Pathog. 2024 Feb 5;20(2):e1011840. doi: 10.1371/journal.ppat.1011840 (PMC10868789; doi:10.1371/journal.ppat.1011840)

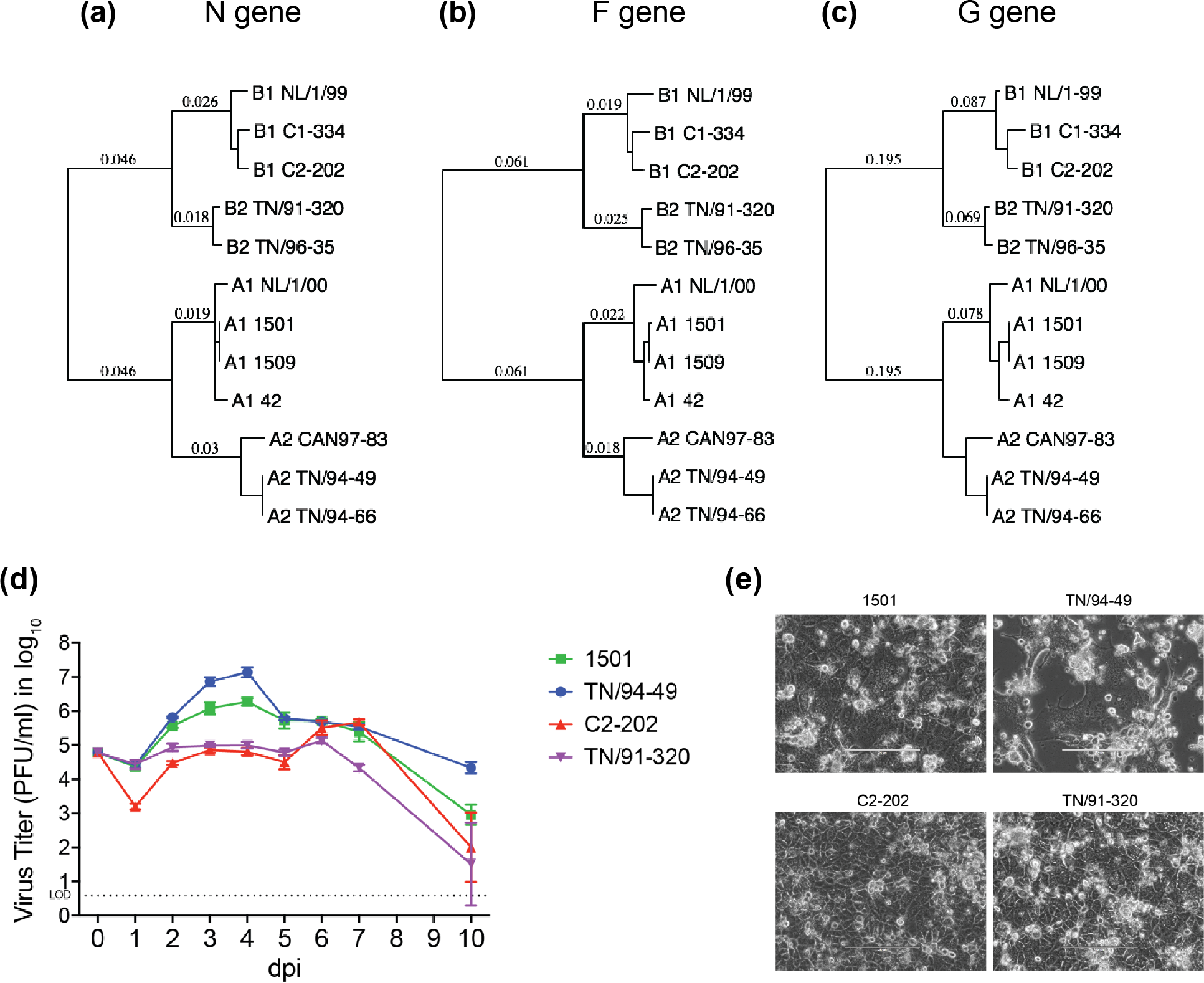

Supplement: S1 Fig — (a-c) Phylogenic trees for nine HMPV clinical isolate strains together with reference strain NL/1/00 (AF371337), NL/1/99 (AY525843), and CAN/97-83 (AY297749) were generated by MacVector 16 software based on the nucleotide sequence of the identified full-length N (a), F (b), or G (c) genes. The ClustalW algorithm was used to align the sequences before creating phylogenic trees with neighbor joining method. Branch lengths are proportional to the inferred amount of nucleotide changes. (d) Growth kinetics of HMPV clinical isolate strains 1501 (A1), TN/94-49 (A2), C2-202 (B1), and TN/91-320 (B2) in LLC-MK2 cells. Cells were inoculated with MOI = 0.1 of each isolate and incubated with growth medium containing 0.5 μg/ml trypsin. Each day, 200 μl of supernatant fluid was collected and snap-frozen, and fresh medium was supplemented. Data were shown as mean with SD from three replicates. I Representative images of HMPV clinical isolates infected LLC-MK2 cells at 5 days post infection. Scale bar 200 μm. (TIF) [file ppat.1011840.s001.tif]

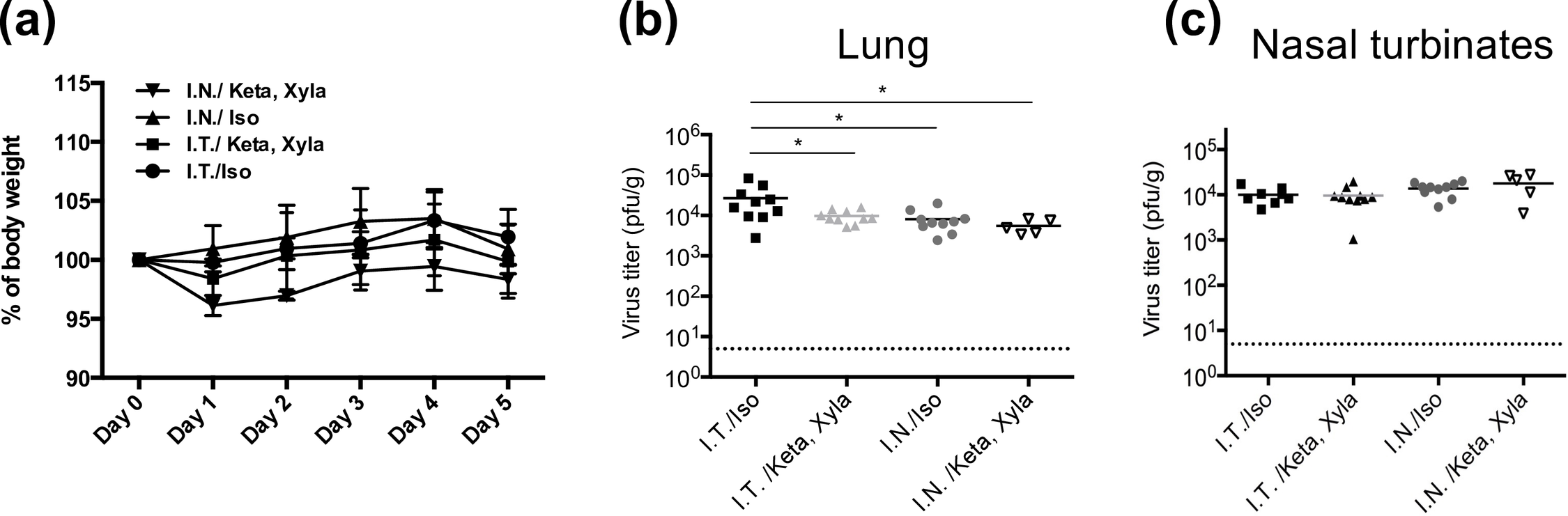

Supplement: S2 Fig — Seven-week-old female B6 mice were anesthezed with inhaled isoflurane (Iso) or injected ketamine/xylazine (Keta, Xyla) and inoculated with 1.0 x 10^6^ PFU HMPV TN/94-49 in 100 μl via I.T. on I.N. route. Weight (a) was monitored daily, and lung (b), and nasal turbinate (c) viral titer determined on D5. * = P<0.05, one-way ANOVA. (TIF) [file ppat.1011840.s002.tif]

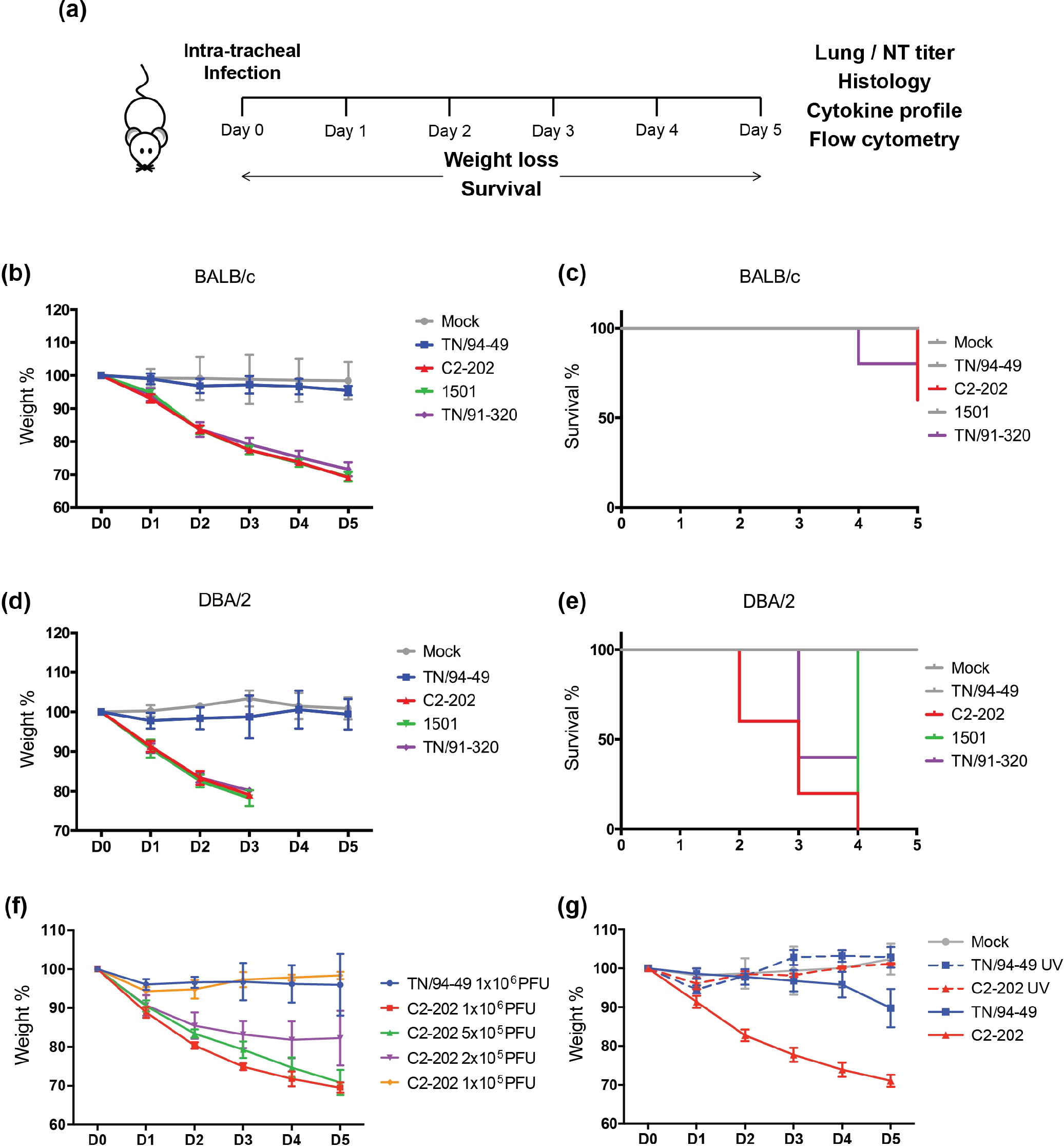

Supplement: S3 Fig — (b,c) Seven-week old female BALB/c mice were infected with 1.0 x 10^6^ PFU HMPV clinical isolates or comparable amount of LLC-MK2 lysates (mock) diluted in 100 μl PBS via I.T. route. Weight loss (b) and survival (c) were monitored daily. N = 5 mice per group. Similar experiments were performed in age-matched female DBA/2 mice (d,e) with 5 mice per group. (f) Dose-dependent weight loss by HMPV clinical isolate C2-202. C57BL/6 mice were infected by varying dosages of C2-202 or 1.0 x 10^6^ PFU TN/94-49 diluted in 100 μl PBS via I.T. route. Data are shown from 5 mice per group except where mice died, leaving fewer. (g) C57BL/6 mice were infected by 1.0 x 10^6^ PFU live or UV-inactivated TN/94-49 and C2-202 via I.T. route. Data are shown from 2–6 mice per group. Schematic created with BioRender.com. (TIF) [file ppat.1011840.s003.tif]

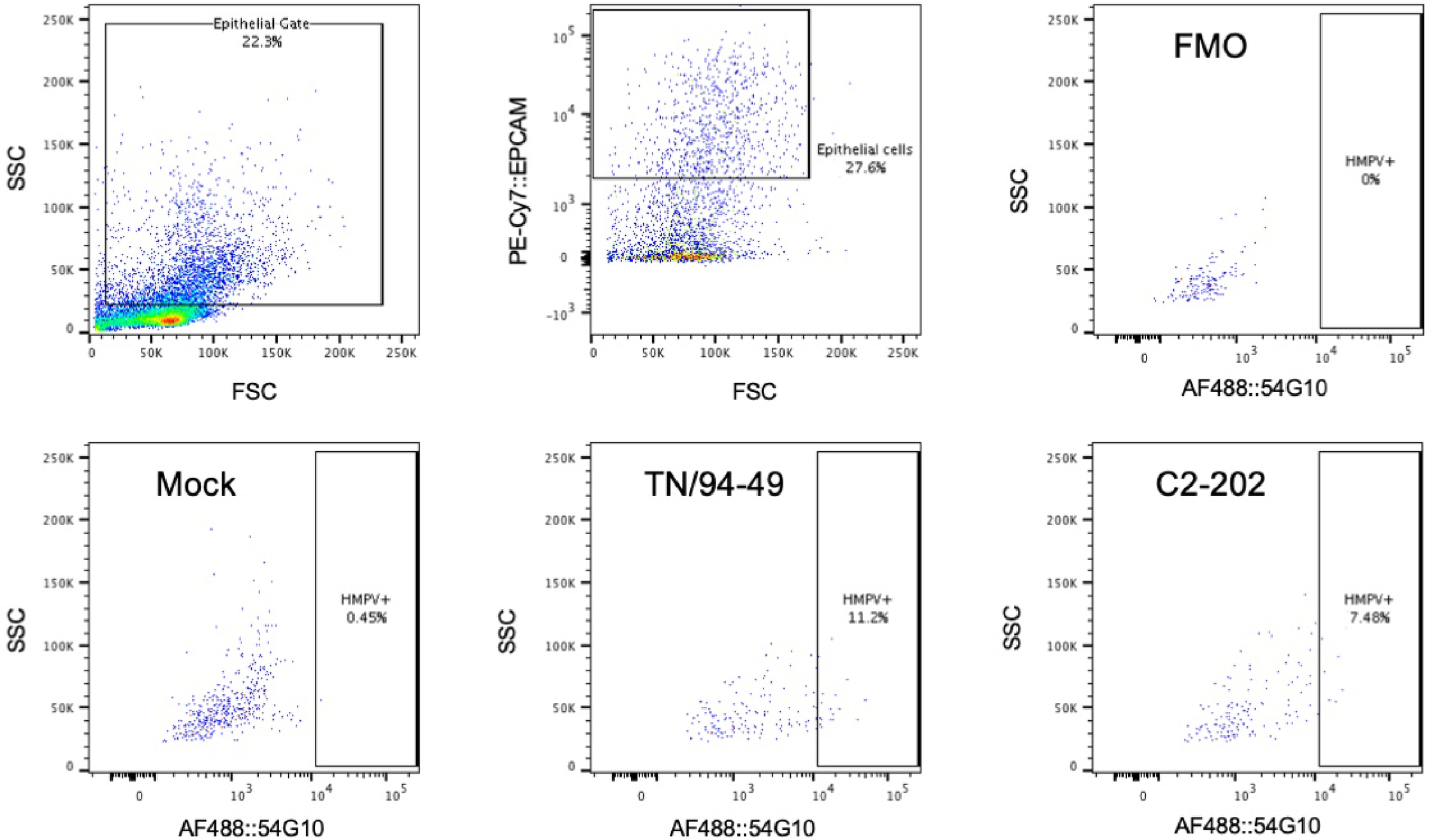

Supplement: S4 Fig — Mice were infected as described and on day 5 post-infection, a single-sell suspension was prepared from lung homogenate. Cells were stained with a monoclonal anti-HMPV F mAb 54G10 and anti-EpCAM Ab. Cells were analyzed by flow cytometry gated by forward and side scatter, and HMPV F+ EpCAM+. Mouse lungs infected by C2-202 had 7.5% HMPV F+ EpCAM+ lung epithelial cells on day 5 p.i. vs. TN/94-49 (11.2%). Representative plots from replicate experiments is shown. (TIF) [file ppat.1011840.s004.tif]

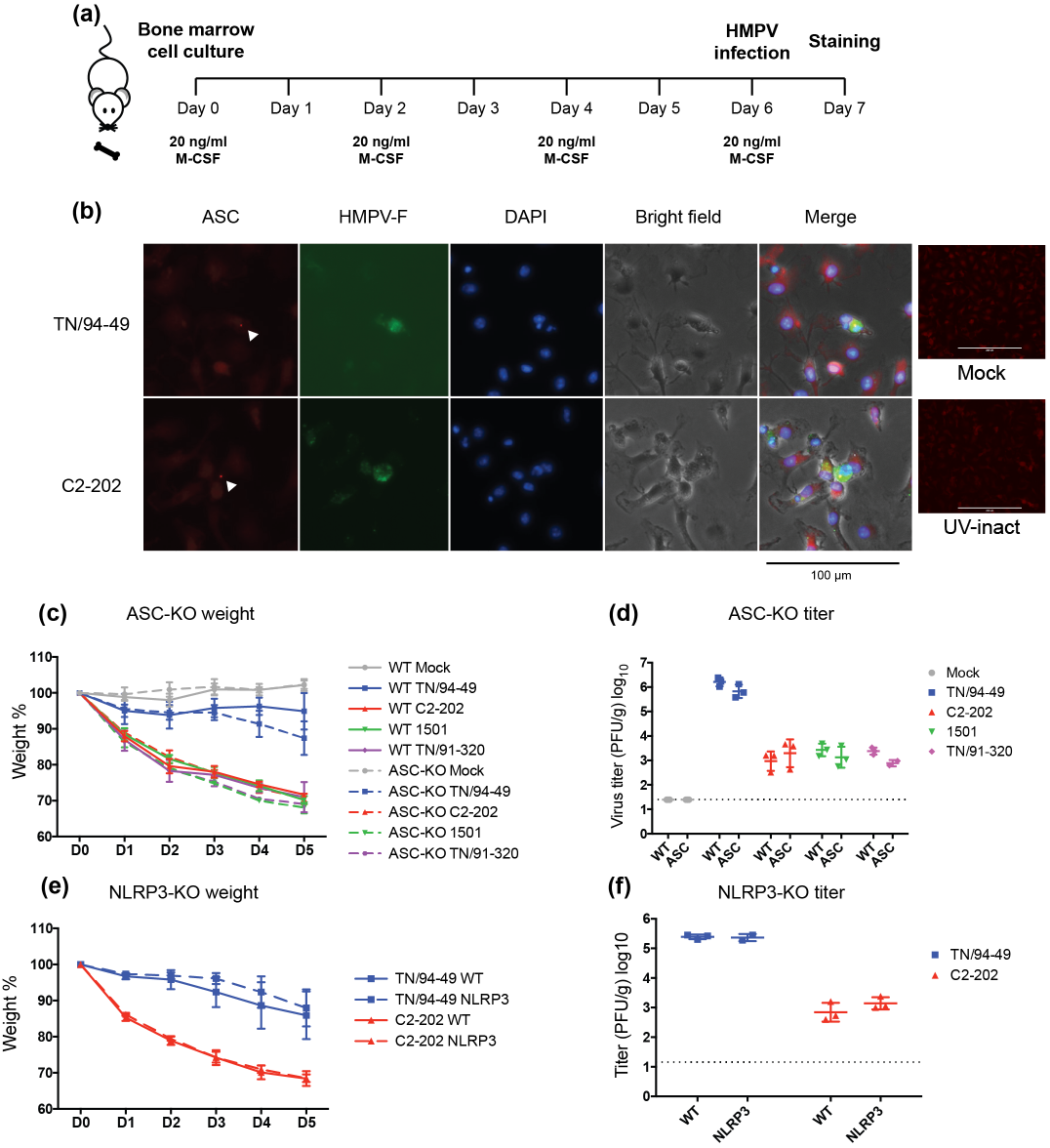

Supplement: S5 Fig — (a) Culture scheme of murine bone marrow-derived macrophages (BMDMs). (b) Immunofluorescent assay on HMPV-infected, mock-infected, or UV-inactivated HMPV infected BMDMs. ASC was labeled with fluorophore AF568 (red), and HMPV-F was labeled with fluorophore AF488 (green). The nucleus was stained by DAPI (blue). The white arrowhead points to the aggregated ASC indicating formation of inflammasome structure. Scale bar 100 μm. (c,d) Weight change and lung titer of HMPV clinical isolates in ASC-KO mice. Female wild type C57BL/6 or ASC-KO mice were infected with 1.0 x 10^6^ PFU HMPV clinical isolates via I.T route. The lungs were harvested 5 days post-infection for titration. Data are shown as mean ±SD from three mice per group. (e,f) Weight change and lung titer of TN/94-49 and C2-202 in NLRP3-KO mice. Age-matched male and female wild type C57BL/6 or NLRP3-KO mice were infected with 1.0 x 10^6^ PFU TN/94-49 or C2-202 via I.T route. The lungs were harvested 5 days post-infection for titration. Data are shown as mean ±SD from 2–3 mice per group. All knock-out mice were co-housed with WT for 2 weeks before experiments. Schematic created with BioRender.com. (PNG) [file ppat.1011840.s005.png]

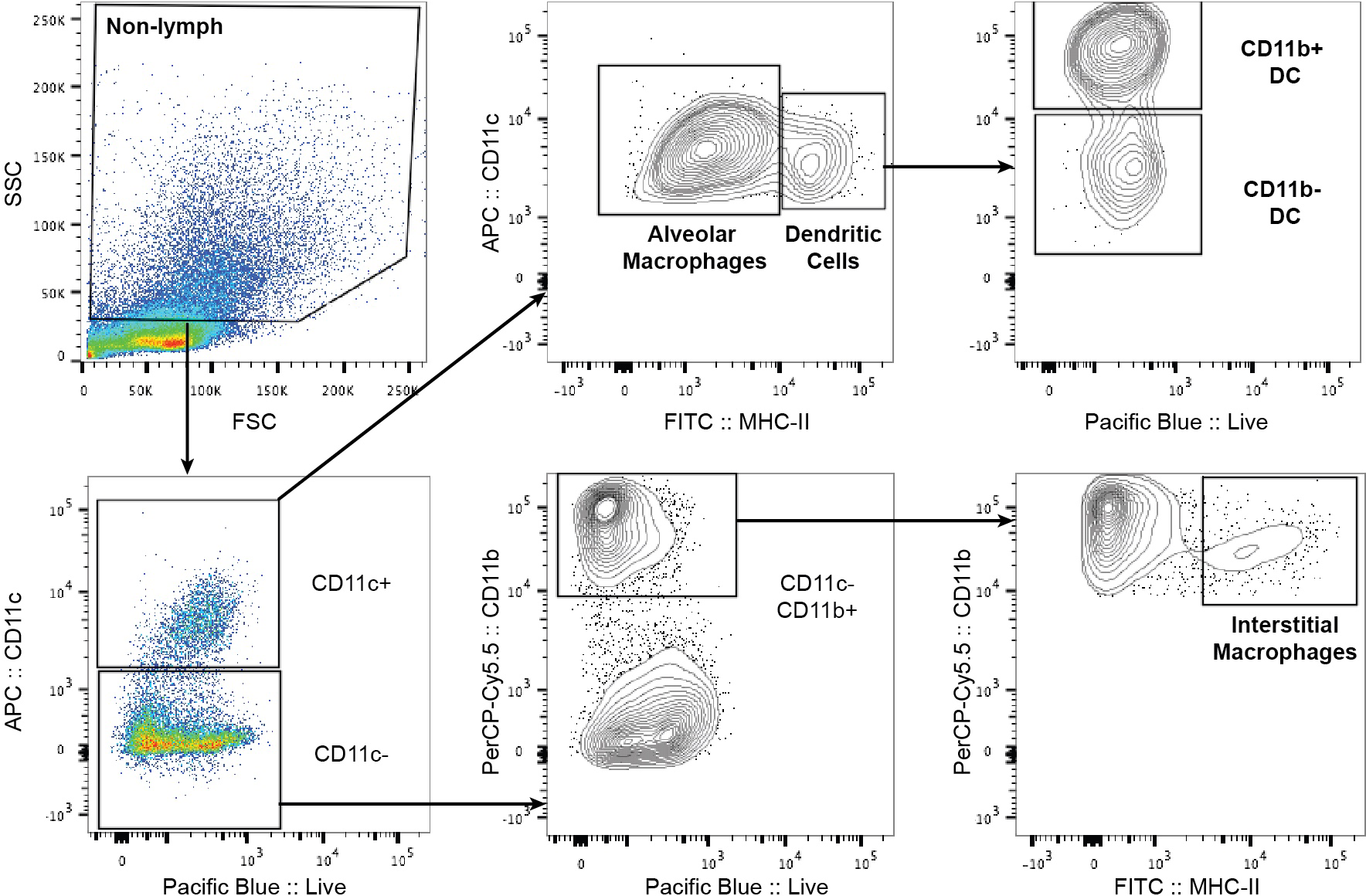

Supplement: S6 Fig — Single cell suspension from lungs of C57BL/6 mice were prepared as described in Methods. The lung cells were treated with Fc-block reagent before staining with surface markers CD11b, CD11c, and MHC-II. Flow cytometric data were analyzed with FlowJo software. The panel shown was gated on single live cells after excluding doublets by forward and aide scatter. (TIF) [file ppat.1011840.s006.tif]

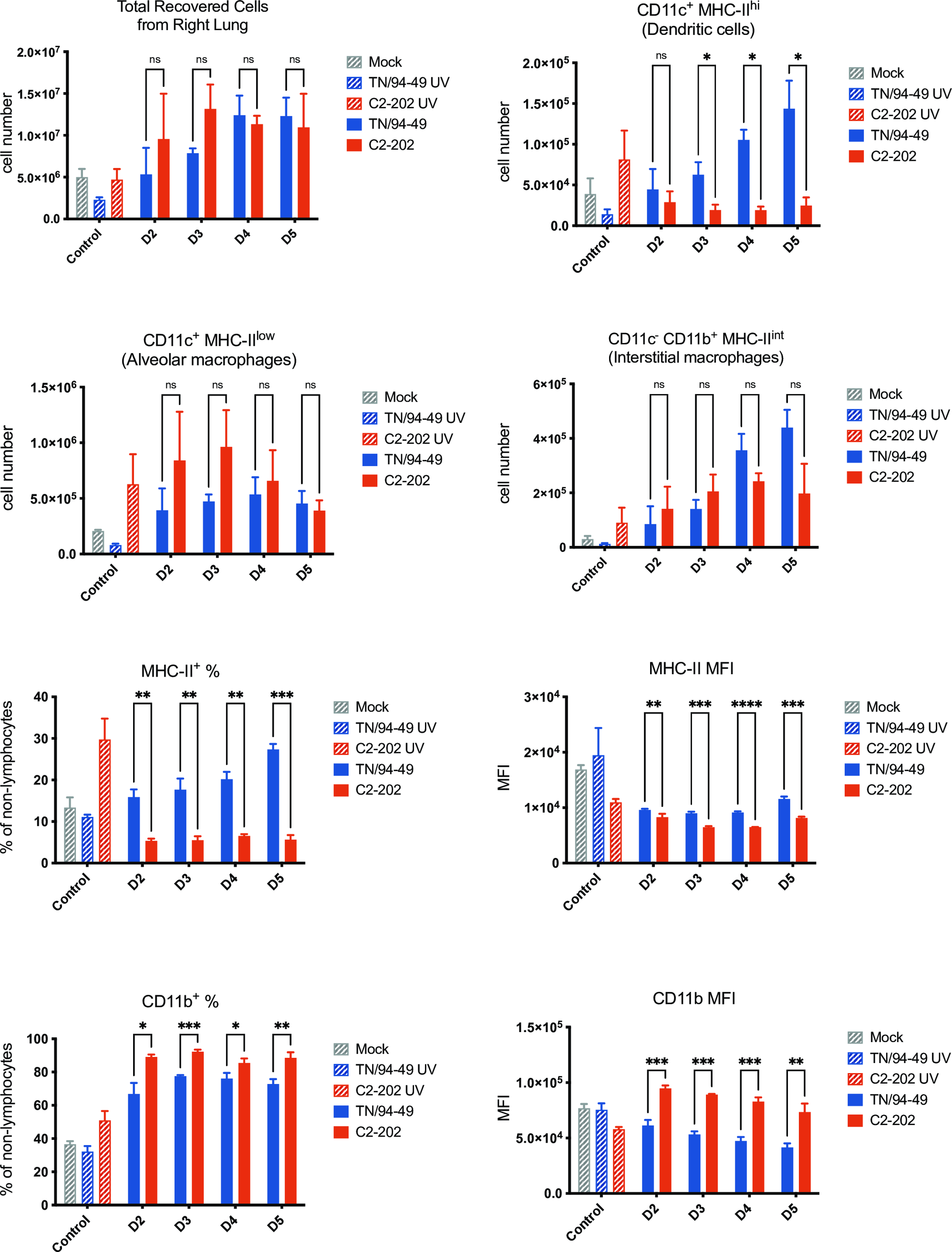

Supplement: S7 Fig — Quantification of flow cytometry analysis on inflammatory cell recruitment following HMPV TN/94-49 and C2-202 infection in a time-course experiment. The number of total recovered cells (a), dendritic cells (b), alveolar macrophages (c), and interstitial macrophages (d) for each time point were shown as mean ±SD from three mice per group. The control group sample were collected at day 5 post infection. The percentage of marker-positive cells in total cells and mean fluorescent intensity (MFI) of MHC-II (e,f) and CD11b (g,h) were also shown as mean ±SD as above. * P<0.05, ** P<0.01, *** P<0.005, ****p<0.001, ns p>0.05, t-test with correction for multiple comparisons. (TIF) [file ppat.1011840.s007.tif]

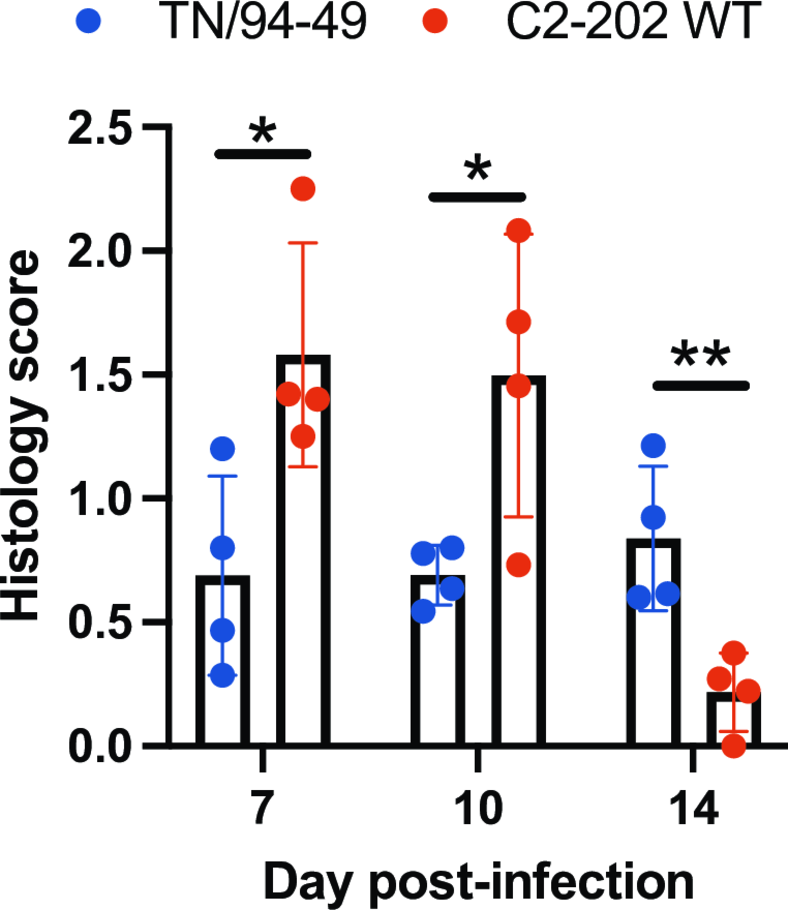

Supplement: S8 Fig — WT B6 mice were infected with 2.5 x 10^5^ of either C2-202 or TN/94-49. Histopathological scores show that the pathogenic isolates C2-202 induced greater histopathological changes on days 7 and 10, with a slight decrease on day 14. * P<0.05, ** P<0.01 by student’s t-test. (TIF) [file ppat.1011840.s008.tif]

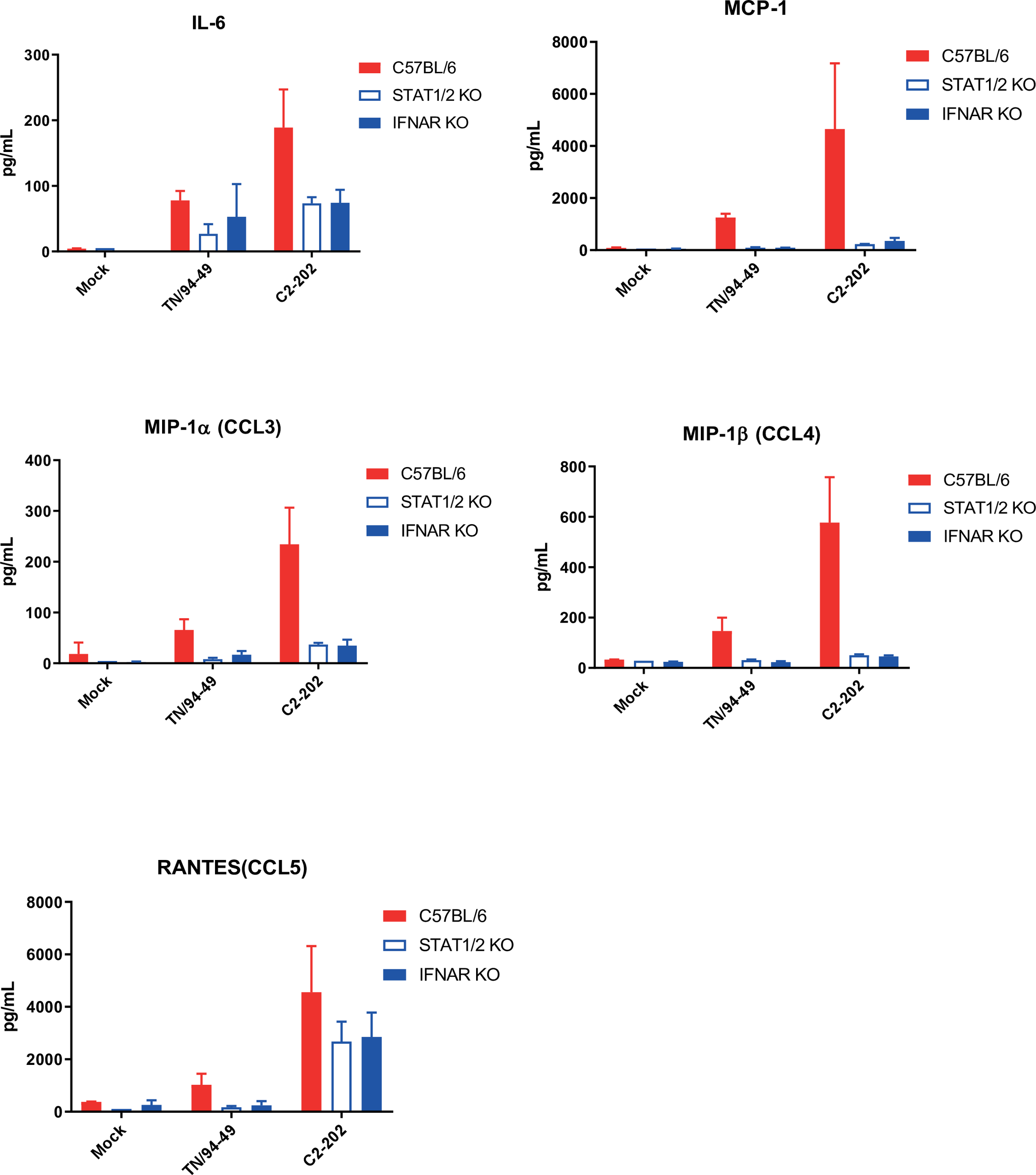

Supplement: S9 Fig — WT B6, IFNAR KO, and STAT-KO mice were infected with 2.5 x 10^5^ of C2-202, euthanized on day 5, and lung homogenate cytokines were measured by ELISA. (TIF) [file ppat.1011840.s009.tif]

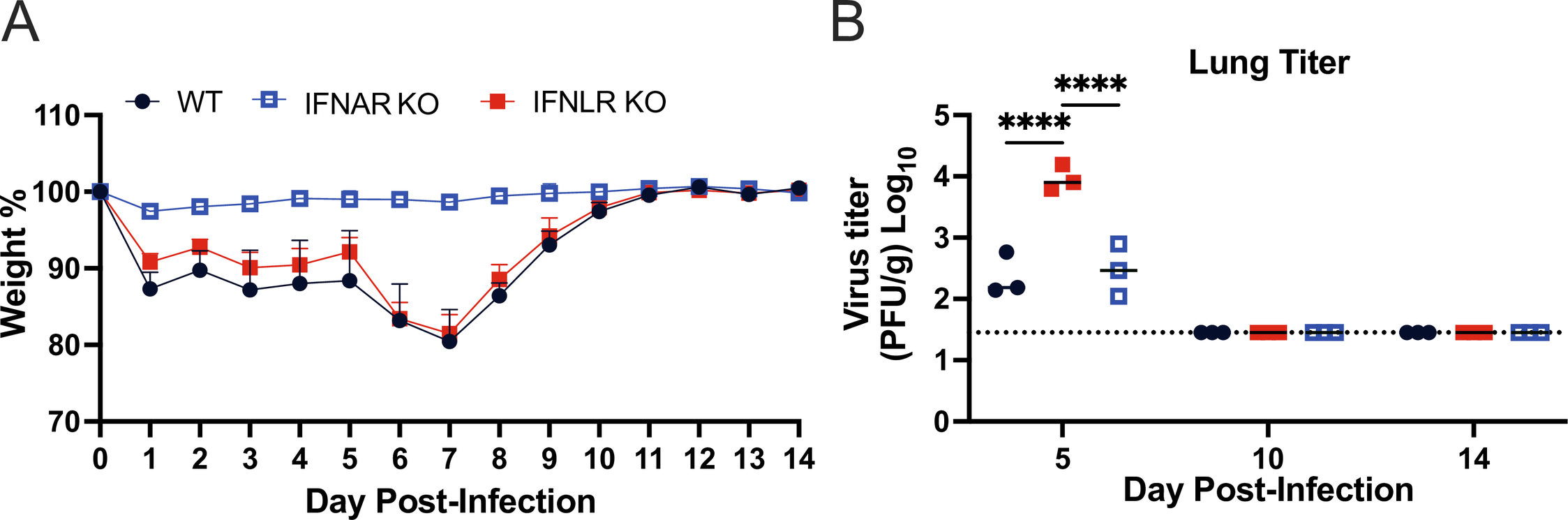

Supplement: S10 Fig — WT, IFNAR KO, and Ifnlr-/- (IFNLR KO) mice were infected with 2.5 x 10^5^ of C2-202 and weighed daily. Subgroups of mice were euthanized on the indicated day post-infection to measure lung virus titer. (TIF) [file ppat.1011840.s010.tif]
