# Supplementary material for: Distinct roles for type I and type III interferons in virulent human metapneumovirus pathogenesis
Source: PLoS Pathog. 2024 Feb 5;20(2):e1011840. doi: 10.1371/journal.ppat.1011840 (PMC10868789; doi:10.1371/journal.ppat.1011840)
Supplement: S1 Table — The mean, maximum, and minimum sequence identity (%) of each paired comparison between any two isolates were listed in the table. Abbreviations: nucleotide (nt), amino acid (aa), maximum identity (max), and minimum identity (min). (DOCX) [file ppat.1011840.s011.docx]

**S1 Table. Sequence identities among HMPV clinical isolates**

|  |  | **N** | **P** | **M** | **F** | **M2-1** | **M2-2** | **SH** | **G** | **L** |
| --- | --- | --- | --- | --- | --- | --- | --- | --- | --- | --- |
| **nt identity %** | **mean** | **90.8** | **87.0** | **90.2** | **89.5** | **90.3** | **90.8** | **79.3** | **70.0** | **89.8** |
|  | max | 100 | 100 | 100 | 100 | 100 | 100 | 100 | 100 | 100 |
|  | min | 85.7 | 79.2 | 84.3 | 83.6 | 84.8 | 85.2 | 67.6 | 54.1 | 84.0 |
| **aa identity %** | **mean** | **97.5** | **90.5** | **98.2** | **96.1** | **96.5** | **93.4** | **72.1** | **51.9** | **96.2** |
|  | max | 100 | 100 | 100 | 100 | 100 | 100 | 100 | 100 | 100 |
|  | min | 95.2 | 83.4 | 96.5 | 93.5 | 93.6 | 88.9 | 55.7 | 29.3 | 93.7 |

The coding sequences of nine HMPV proteins of the nine clinical isolates were aligned in MacVector 16 software with ClustalW algorithm. The mean, maximum, and minimum sequence identity (%) of each paired comparison between any two isolates were listed in the table. Abbreviations: nucleotide (nt), amino acid (aa), maximum identity (max), and minimum identity (min).
